# 5G SLAM Using the Clustering and Assignment Approach with Diffuse Multipath

**DOI:** 10.3390/s20164656

**Published:** 2020-08-18

**Authors:** Yu Ge, Fuxi Wen, Hyowon Kim, Meifang Zhu, Fan Jiang, Sunwoo Kim, Lennart Svensson, Henk Wymeersch

**Affiliations:** 1 Department of Electrical Engineering, Chalmers University of Technology, 412 96 Gothenburg, Sweden; fuxi@chalmers.se (F.W.); fanji@chalmers.se (F.J.); lennart.svensson@chalmers.se (L.S.); henkw@chalmers.se (H.W.); 2 Department of Electronic Engineering, Hanyang University, Seoul 04763, Korea; khw870511@hanyang.ac.kr (H.K.); remero@hanyang.ac.kr (S.K.); 3 Department of Electrical and Information Technology, Lund University, 221 00 Lund, Sweden; meifang.zhu@eit.lth.se

**Keywords:** mmWave single base station SLAM, diffuse multipath, clustering

## Abstract

5G communication systems operating above 24 GHz have promising properties for user localization and environment mapping. Existing studies have either relied on simplified abstract models of the signal propagation and the measurements, or are based on direct positioning approaches, which directly map the received waveform to a position. In this study, we consider an intermediate approach, which consists of four phases—downlink data transmission, multi-dimensional channel estimation, channel parameter clustering, and simultaneous localization and mapping (SLAM) based on a novel likelihood function. This approach can decompose the problem into simpler steps, thus leading to lower complexity. At the same time, by considering an end-to-end processing chain, we are accounting for a wide variety of practical impairments. Simulation results demonstrate the efficacy of the proposed approach.

## 1. Introduction

Progressive generations of cellular communication systems have a common property—each generation relies on a larger bandwidth and higher carrier frequency than the previous one. This is particularly noticeable in 5G, where in Frequency Range 2 (FR2), carriers above 24 GHz are utilized in combination with contiguous bandwidths of up to 400 MHz. Early commercial deployments in the US have demonstrated data rates beyond 1 Gbps [1]. The same increases in bandwidth and carrier frequency also have direct benefits for positioning [2]. This has led to intense research activities into 5G positioning as well as a dedicated study item in 3GPP. The final report of this study in 3GPP [3] revealed that by using both delay and angle measurements, it is possible to satisfy commercial positioning performance requirements. Beyond positioning, the sparse nature of the channel at higher carriers allows the receiver to resolve the multipath components, which transforms them from foe to friend [4,5]. In particular, the geometric nature of the channel at mmWave frequencies leads to a relation between the multipath parameters and the physical environment, as the parameters are related to the location of the user (UE) with respect to the base station (BS) and the propagation environment. The simultaneous localization and mapping problem is then invoked to invert multipath parameters into geometric information that determines the user’s position and the locations of objects, based on the signal from a single base station. Several papers have addressed this problem, exploiting both line-of-sight (LOS) and non-LOS (NLOS) paths for position estimation, synchronization, and mapping in mmWave multiple-input multiple-output (MIMO) [6,7,8] and mmWave multiple-input single-output (MISO) [9,10] contexts. However, this inversion is challenging due to a number of reasons: (i) estimation of the channel parameters involves solving a high-dimensional problem; (ii) the association between the objects in the environment and the measurements is not available; (iii) an object may give rise to multiple measurements, due to diffuse multipath; (iv) the measurements per object are not clustered. We will now treat each of these four challenges in turn.

Channel estimation for mmWave is a rich research area, which we cannot cover in detail here. Rather, we categorize these methods as search-based and search-free. Search-based methods, such as those using maximum likelihood (ML) [11] and compressed sensing (CS) techniques [12,13], require an exhaustive search in the high-dimensional space of channel parameters, which entails high complexity. On the other-hand, search-free methods [14], such as matrix or tensor decomposition based subspace methods, directly provide estimates of the channel parameters [15] or rely on low-dimensional search [16], thus avoiding the need for high-dimensional optimization. An important challenge of channel estimation for SLAM is that the different dimensions (angles of arrival and departure, delays and gains) should be correctly matched.

The unknown association between measurements and objects is a common problem in SLAM, and powerful methods to address it can be found in the literature [17,18]. SLAM with radio-based measurements has been considered in the context of ultra-wideband (UWB) communication [19,20] using only distance measurements, which is referred to multipath-assisted SLAM, or channel SLAM. Focusing on the application of SLAM in a 5G mmWave context (which we designate ’5G SLAM’), message passing-based estimators were introduced in References [7,21], based on the concept of non-parametric belief propagation, without the data association (DA). Extension of such methods to include the hidden DAs is possible, following the approaches from Reference [22]. In Reference [23], the probability hypothesis density (PHD) filter, which is a random-finite-set filter, was used to solve the 5G SLAM problem, considering only one measurement per object. In Reference [24], a more powerful random-finite-set filter, Poisson multi-Bernoulli mixture (PMBM) filter, was used, which enumerates all possible DAs.

**Figure 1:**
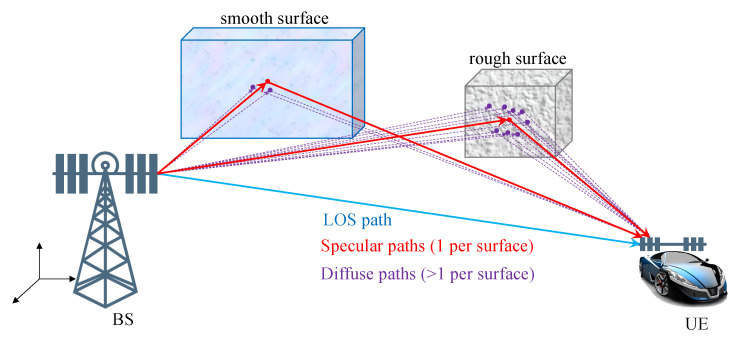
In mmWave simultaneous localization and mapping (SLAM) applications, each object can give rise to a specular path as well as multiple diffuse paths. The number and spread of these diffuse paths depend on the roughness of the object. At the receiver side, diffuse paths from an object have similar delays and angles, so that they can only be resolved with sufficient bandwidth and number of antennas.

Multiple measurements per object, while common in the SLAM and extended object tracking literature [25], have been generally ignored in the above 5G SLAM works. The multiple measurements per object are caused by diffuse multipath due to object roughness with respect to the wavelength, depicted in Figure 1. In Reference [5], diffuse multipath is seen as a perturbation, leading to false measurements. In Reference [26], exploitation of the diffuse multipath in radar is proposed by means of diffuse multipath statistics. In Reference [27], surface roughness was considered in radar applications, modeled as a number of sub-reflectors, in an environment with known wall geometry. A similar model with random sub-reflectors was evaluated in Reference [28], where the estimated diffuse paths were used for positioning and mapping, using a simple geometric approach.

Finally, when measurements from an object are not clustered, the unknown grouping can be considered within the SLAM filter [29,30,31], though this comes at a high computational cost. In Reference [28], a K-means clustering was utilized, but this requires a priori knowledge of the number of clusters. In Reference [32], K-power-means was proposed, as well as several criteria to decide the number of clusters. In Reference [24], the perfect clustering was assumed.

In this paper, we address the aforementioned challenges, building on the extensive literature in each of the above research areas in order to provide an end-to-end framework for SLAM harnessing diffuse multipath. The proposed end-to-end framework provides a general approach for user localization and environment mapping in 5G downlink transmissions from a single BS. Therefore, the purposed framework can be utilized in many application areas, including personal navigation [4], localization of cars and robots  [33], smart homes [34], indoor location analysis [35], immersive customer experiences [36], location-aided communication [37], personal radar [38], to name but a few. Moreover, the proposed framework can form a foundation for future Beyond 5G and 6G localization and sensing approaches [39]. Our framework is built on a layered approach, comprising three main parts (channel estimation, clustering, and SLAM), which are evaluated separately and end-to-end. The main contributions of this paper are as follows:

- The description of an end-to-end framework for SLAM harnessing diffuse multipath and its performance evaluation.
- The evaluation of clustering and assignment methods, which is suitable for estimated channel parameters under both specular and diffuse multipath, as well as a method to utilize the estimated channel gains for improving the clustering in the 5G SLAM problem.
- The extension of the 5G SLAM likelihood function, in order to harness both specular and diffuse multipath components and to classify different object types according to their roughness, while accounting for clustering errors.

The novelty of the proposed approach compared to the existing random finite set (RFS) based 5G SLAM work [23,40] is three-fold—first of all, References [23,40] did not use a real channel estimator, which makes the problem easier. Secondly, they assumed at most one measurement from an object, which is not the real case. Finally, the PHD filter is not optimal, which does not contain the enumeration of the different data associations. In Reference [24], although the measurements are from the ESPRIT channel estimator and the diffuse multipath is considered, the channel gain is still ignored and the channel estimation results are assumed to be well grouped based on the source. In the current paper, we study the whole framework, from downlink signals to SLAM filter. We also fully use the information given by the channel estimator, including diffuse multipath and channel gain. The PMBM filter is used, which is optimal and enumerates all possible data associations.

The remainder of this paper is structured as follows. The system model is described in Section 2, including the signal model, environment model, sensor, and measurement model. The end-to-end framework is then presented in Section 3, specifying the components that will be detailed in the subsequent sections, starting with channel estimation in Section 4, clustering in Section 5, and the novel likelihood in Section 6. Simulation results are presented in Section 7, followed by our conclusions in Section 8. The paper also contains several appendices describing the geometric expressions of the channel parameters, as well as the details of the SLAM method.

### Notations

Scalars (e.g., *x*) are denoted in italic, vectors (e.g., x) in bold, matrices (e.g., X) in bold capital letters, sets (e.g., X) in calligraphic, tensors (e.g., X) in bold calligraphic. Transpose and Hermetian are denoted by $\cdot^{T}$ and $\cdot^{H}$, and $ȷ=\sqrt{-1}$. Furthermore, $\left|\;\cdot \;\right|$ denotes the absolute value of a scalar, or the cardinality of a set; $∥\;\cdot \;∥$ denotes the Euclidean norm of a vector. A Gaussian density with mean μ and covariance Σ, evaluated in value x is denoted by N(x;μ,Σ). Finally, the notations of important variables are summarized in Table 1.

## 2. System Model

In this section, we describe the basic models of the user, the environment, the propagation channel, and the observed waveform. We use the 5G positioning reference signals (PRS) [41] as pilot signals. These signals are broadcast by the BS, and used for positioning according to the 3GPP standards [3,42]. Since the signals are broadcast, there is no multi-user issue or interference, so that we only focus on a single UE, where the proposed algorithms can be assumed to be executed per UE.

### 2.1. User Model

In this paper, we only consider the single-user scenario, so the cooperation between different users is beyond the scope of this paper. The dynamic state of the user at time step *k* is denoted by $s_{k}$, which contains the position of the user $x_{\mathrm{UE},k}={\left[x_{k},y_{k},z_{k}\right]}^{T}$, heading $\varpi_{k}$, translation speed $\zeta_{k}$, turn rate $\varrho_{k}$ and clock bias $B_{k}$. The dynamic model of $s_{k}$ is

(1) $$s_{k}=v\left(s_{k-1}\right)+q_{k},$$

where v(·) denotes a known transition function; $q_{k}$ is the process noise, modeled as a zero-mean Gaussian with known covariance $Q_{k}$. The above dynamic model can be expressed, equivalently, in terms of a transition density $f\left(s_{k}|s_{k-1}\right)=N\left(s_{k};v\left(s_{k-1}\right),Q_{k}\right)$.

### 2.2. Environment Model

**Figure 2:**
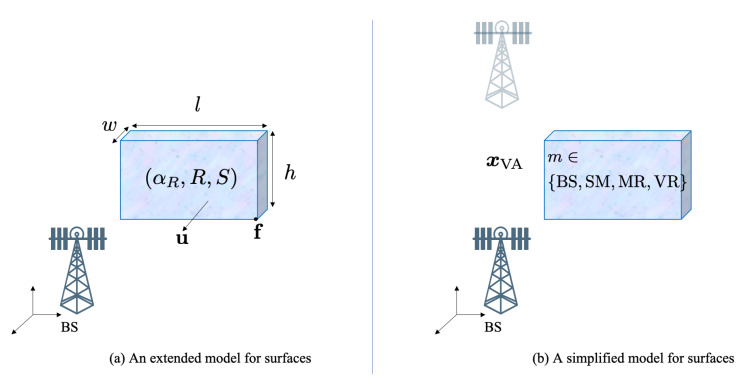
Two different surface models: (**a**) shows a high-dimensional model, while (**b**) is a compact model that expresses the location of the surface via a virtual anchor.

We consider an environment with a few landmarks. There is a fixed and known BS, located at $x_{\mathrm{BS}}\in R^{3}$. The other unknown landmarks are modeled as different types of surfaces (see Figure 2). A surface can be described with an extended state comprising:

- A point f on a corner of the surface and a vector u normal to the surface.
- The size of the surface in length *l* and height *h*. The width *w* is not relevant.
- The smoothness of the surface, denoted by $\alpha_{R}\geq 0$.
- The scattering attenuation 1≥S≥0 and reflection attenuation 1≥R≥0, with $R^{2}+S^{2}\leq 1$, in which the remaining power is absorbed in the surface.

To avoid dealing with such a high-dimensional surface state description, we use a simplified state, containing a fixed virtual anchor (VA) with location $x_{\mathrm{VA}}\in R^{3}$, which is the reflection of the BS with respect to the surface, that is,

(2) $$\begin{array}{c} x_{\mathrm{VA}}=\left(I-2uu^{T}\right)x_{\mathrm{BS}}+2f^{T}uu. \end{array}$$

Note that the VA location is surface-specific. We only consider 3 different types of surfaces: smooth surface (SM, with S=0, R=0.8, $\alpha_{R}=100$), medium rough surface (MR, with $S=0.4,R=0.6,\alpha_{R}=4$) and very rough surface (VR, with $S=0.8,R=0,\alpha_{R}=0$). Signals from the base station can only be reflected via SM to the receiver; while they can be reflected and diffused via MR to the receiver; whereas VR can only diffuse signals. This allows us to have a compact state representation, with the landmark state $x={\left[x_{\mathrm{LM}}^{T},m\right]}^{T}$, where m∈{BS,SM,MR,VR} and $x_{\mathrm{LM}}=x_{\mathrm{BS}}$ for m=BS, while $x_{\mathrm{LM}}=x_{\mathrm{VA}}$, for m≠BS [24].

### 2.3. Channel Model

At time step *k*, the channel from the BS to the user in the frequency domain is given by  [11]:

(3) $$\begin{array}{c} H_{k}\left(f,\theta ,\phi \right)=\sum_{i=0}^{I_{k}-1}\sum_{l=0}^{L_{k}^{i}-1}g_{k}^{i,l}\delta \left(\theta -\theta_{k}^{i,l}\right)\delta \left(\phi -\phi_{k}^{i,l}\right)e^{-ȷ2\pi f\tau_{k}^{i,l}}, \end{array}$$

where δ(·) is the delta Dirac function, *f* is the frequency, θ is the angle of arrival (AOA), ϕ is the angle of departure (AOD), $I_{k}$ is the number of landmarks in the environment (i=0 represents the BS); $L_{k}^{i}$ is the number of paths from each landmark (l=0 represents the specular path). For each surface, there is at most one deterministic specular component among the paths, while all remaining paths are diffuse components and thus random [28]. We denote the total number of paths by $P_{k}=\sum_{i=1}^{I_{k}}L_{k}^{i}$. Each path i,l can be described by a complex gain $g_{k}^{i,l}$, a time of arrival (TOA) $\tau_{k}^{i,l}$, an AOA pair $\theta_{k}^{i,l}$ in both azimuth and elevation, and an AOD pair $\phi_{k}^{i,l}$ in both azimuth and elevation. The generative model for each of these parameters is described as

- *LOS path:* When i=0, $L_{k}^{i}=1$, the gain has uniform phase and has power
  (4) $$\begin{array}{c} {\left|g_{k}^{0,0}\right|}^{2}=\frac{\lambda^{2}}{{\left(4\pi \right)}^{2}{∥x_{\mathrm{UE}}-x_{\mathrm{BS}}∥}^{2}}, \end{array}$$
  where λ is the wavelength, and the TOA, AOA, and AOD follow the geometric relations between the BS and the UE. They are given in Appendix A.
- *Specular path from surface i:* For i>0, l=0, the point of incidence on the *i*-th surface (denoted by $L_{i}$ with virtual anchor $x_{\mathrm{VA},i}$) is the intersection of the surface $L_{i}$ and the line between the *i*-th virtual anchor $x_{\mathrm{VA},i}$ and the UE $x_{\mathrm{UE}}$. The specular path gain has uniform phase and power
  (5) $$\begin{array}{c} {\left|g_{k}^{i>0,0}\right|}^{2}=\frac{\lambda^{2}R^{2}}{{\left(4\pi \right)}^{2}{∥x_{\mathrm{UE}}-x_{\mathrm{VA},i}∥}^{2}}. \end{array}$$
  The TOA, AOA, and AOD follow the relative position of the UE, BS, and the incidence point on the surface. They are given in Appendix A.
- *Diffuse paths from surface i:* For i>0, l>0, the number of paths per surface and their spread in angle and delay, as well as the channel gains, depend on the roughness of that surface. These paths can be interpreted as coming from random points $x_{k}^{i,l}$ on the surface, with a spatial distribution $p_{i}\left(x_{\mathrm{sc}}|x_{\mathrm{UE}},x_{\mathrm{BS}}\right)$ that depends on the roughness, where $x_{\mathrm{sc}}$ is the random variable that describes the position of the diffuse point. The diffuse points are generated from the distribution $x_{k}^{i,l}∼p_{i}\left(x_{\mathrm{sc}}|x_{\mathrm{UE}},x_{\mathrm{BS}}\right)$ ([43],Chapter 3)
  (6) $$\begin{array}{cc} p_{i}\left(x_{\mathrm{sc}}|x_{\mathrm{UE}},x_{\mathrm{BS}}\right) & \propto \left\{\begin{array}{cc} \frac{{\left(1+\cos\;\nu_{1}\left(x_{\mathrm{sc}}\right)\right)}^{\alpha_{R}}}{2}\frac{|\cos\;\nu_{2}\left(x_{\mathrm{sc}}\right)||\cos\;\nu_{3}\left(x_{\mathrm{sc}}\right)|}{∥x_{\mathrm{sc}}-x_{\mathrm{BS}}{∥}^{2}{∥x_{\mathrm{sc}}-x_{\mathrm{UE}}∥}^{2}} & x_{\mathrm{sc}}\in L_{i}\\ 0 & \mathrm{else}, \end{array}\right. \end{array}$$
  where $L_{i}\subset R^{3}$ denotes the set of points that make the *i*-th surface, $\nu_{1}\left(x_{\mathrm{sc}}\right)$ is the deviation of the scattering angle with respect to the angle of the specular path (i.e,. $\nu_{1}\left(x_{\mathrm{sc}}\right)=0$ when $x_{\mathrm{sc}}$ is the incidence point of the specular path), $\nu_{2}\left(x_{\mathrm{sc}}\right)$ is the angle between the impinging ray (i.e., from the transmitter to $x_{\mathrm{sc}}$) and the surface normal, and $\nu_{3}\left(x_{\mathrm{sc}}\right)$ is the angle between the departing ray and the surface normal. The diffuse paths have uniform and independent phases and equal power
  (7) $$\begin{array}{c} {\left|g_{k}^{i>0,l>0}\right|}^{2}=\frac{\lambda^{2}S^{2}\left(\alpha_{R}+1\right)}{{\left(4\pi \right)}^{2}\left(L_{k}^{i}-1\right)\left(1-2^{-(\alpha_{R}+1)}\right)\left(\alpha_{R}+2\right){∥x_{\mathrm{UE}}-x_{\mathrm{VA},i}∥}^{2}}. \end{array}$$
  The locations of the diffuse points $x_{k}^{i,l}$ fully determine their corresponding TOA, AOA, and AOD, provided in Appendix A.

### 2.4. Signal Model

We will assume a transmitter and receiver with uniform rectangular arrays (URAs) with $M_{T}=M_{1}\times M_{2}$ (i.e., $M_{1}$ columns and $M_{2}$ rows) and $M_{R}=M_{3}\times M_{4}$ elements, respectively. The corresponding steering vectors are

(8) $$\begin{array}{cc} a_{T}\left(\phi =\left[\phi_{az},\phi_{\mathrm{el}}\right]\right) & =a_{c}\left(\phi \right)⊗a_{r}\left(\phi \right) \end{array}$$

(9) $$\begin{array}{cc} {}[a_{c}{\left(\phi \right)]}_{m_{c}} & =\exp\left(ȷ\pi m_{c}\sin\left(\phi_{\mathrm{el}}\right)\right),\;\;m_{c}\in \left\{0,\ldots ,M_{2}-1\right\} \end{array}$$

(10) $$\begin{array}{cc} {}[a_{r}{\left(\phi \right)]}_{m_{r}} & =\exp\left(ȷ\pi m_{r}\cos\left(\phi_{\mathrm{el}}\right)\sin\left(\phi_{\mathrm{az}}\right)\right),\;\;m_{r}\in \left\{0,\ldots ,M_{1}-1\right\} \end{array}$$

and

(11) $$\begin{array}{cc} a_{R}\left(\theta =\left[\theta_{\mathrm{az}},\theta_{\mathrm{el}}\right]\right) & =a_{c}\left(\theta \right)⊗a_{r}\left(\theta \right) \end{array}$$

(12) $$\begin{array}{cc} {}[a_{c}{\left(\theta \right)]}_{m_{c}} & =\exp\left(ȷ\pi m_{c}\sin\left(\theta_{\mathrm{el}}\right)\right),\;\;m_{c}\in \left\{0,\ldots ,M_{4}-1\right\} \end{array}$$

(13) $$\begin{array}{cc} {}[a_{r}{\left(\theta \right)]}_{m_{r}} & =\exp\left(ȷ\pi m_{r}\cos\left(\theta_{\mathrm{el}}\right)\sin\left(\theta_{\mathrm{az}}\right)\right),\;\;m_{r}\in \left\{0,\ldots ,M_{3}-1\right\}, \end{array}$$

in which ⊗ is the Kronecker product.

In addition, we consider OFDM transmission with $M_{S}=M_{5}$ subcarriers and subcarrier spacing Δf. The received signal at subcarrier *s* at time step *k* is then [44]

(14) $$\begin{array}{c} Y_{s,k}=\underset{H_{s}}{\underbrace{\sum_{i=0}^{I_{k}-1}\sum_{l=0}^{L_{k}^{i}-1}g_{k}^{i,l}a_{R}\left(\theta_{k}^{i,l}\right)a_{T}^{H}\left(\phi_{k}^{i,l}\right)e^{-ȷ2\pi s\Delta f\tau_{k}^{i,l}}}}S_{s}+N_{s,k}, \end{array}$$

where $S_{s}\in C^{M_{T}\times T}$ is the pilot signal over subcarrier *s* (spanning *T* OFDM symbols), $N_{s,k}$ is white Gaussian noise with $\mathrm{vec}\left(N_{s,k}\right)∼\mathrm{CN}\left(0,\sigma^{2}I_{M_{R}T}\right)$, and $H_{s}$ is the channel frequency response,

## 3. Methodology and End-to-End Framework

**Figure 3:**
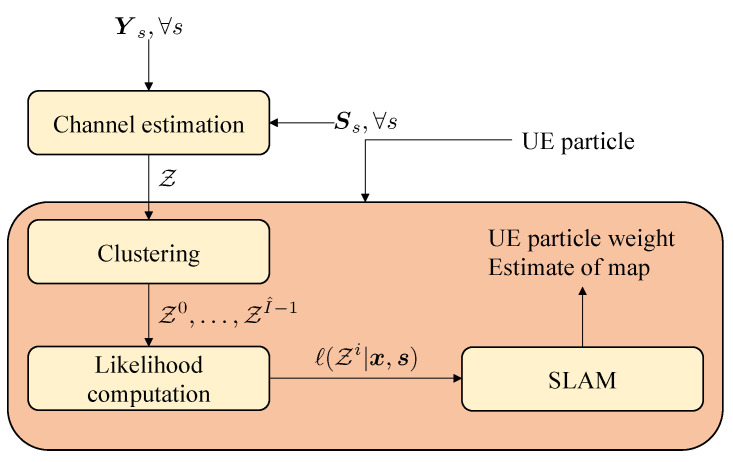
Proposed layered approach for SLAM from the observations $Y_{s}$. The time index *k* is omitted.

Using the raw measurements $Y_{s}$ directly in SLAM is challenging, due to the high dimensionality of the measurement, the complex nonlinear relation to the user and landmark states, and the fact that not all paths may be resolvable, due to a limited number of transmitter and receiver antennas and bandwidth. While such direct localization has performance benefits [45,46,47], we instead consider a *layered approach*, visualized in Figure 3, comprising the following steps after downlink transmission of the signals $Y_{s}$.

1. First of all, channel estimation is performed to recover the channel parameters (angles, delays, gains). Due to the finite resolution at the receiver side, not all paths are resolvable. Hence, the number of estimated paths (denoted by ${\hat{P}}_{k}$) will be much smaller than $I_{k}\times L_{k}^{i}$. The channel estimator thus provides a set of channel parameter estimates $Z_{k}$ at time *k*, $Z_{k}=\left\{z_{k}^{0},z_{k}^{1},\cdots ,z_{k}^{{\hat{P}}_{k}-1}\right\}$. Each element $z_{k}^{p}\in Z_{k}$ is either a clutter, which is caused by noise peaks that are detected as paths during channel estimation, with clutter intensity c(z) or follows
  (15) $$\begin{array}{cc} z_{k}^{p} & ={\left[\tau_{k}^{p},{\left(\theta_{k}^{p}\right)}^{T},{\left(\phi_{k}^{p}\right)}^{T},g_{k}^{p}\right]}^{T}+w_{k}^{p} \end{array}$$
  (16) $$\begin{array}{cc} \; & =h\left(x_{k}^{p},s_{k}\right)+w_{k}^{p}, \end{array}$$
  where $w_{k}^{p}$ denotes the measurement noise; $x_{k}^{p}$ is $x_{\mathrm{BS}}$ for LOS, the incidence point of the deterministic specular components, or a (random) point on the surface for a NLOS component. We recall that the underlying geometric relation $h(x_{k}^{p},s_{k})$ can be found in Appendix A. We describe the channel estimator in Section 4.
2. After channel estimation, we group the unordered elements in $Z_{k}$ in clusters $Z_{k}^{i}$, where each cluster should correspond to one landmark. This removes the need to consider all possible partitions of the measurements in the SLAM method, drastically reducing overall complexity. Clustering is challenging as measurement clusters may be non-convex. In addition, diffuse paths may be far away from the specular paths, leading to possible miss-classifications. The proposed clustering method is described in Section 5.
3. Finally, after clustering, the SLAM method requires a likelihood function that expresses the statistical relation between the state and the clustered measurements, $\ell (Z_{k}^{i}|x,s_{k})$. The SLAM method is deferred to Appendix B, while in the main text we focus on the proposed likelihood function in Section 6. The SLAM filter follows a Rao-Blackwellized approach, where we use a set of particles (indexed by *n*) to represent the user state, and use PMBM densities conditioned on each particle to represent the map. Clustering and likelihood computation are conditioned in the user state and are thus performed per particle.

## 4. Channel Estimation

In this section, the ESPRIT channel estimator is introduced.

### 4.1. Background

The standard formulation of the channel estimation problem is an ML problem, where

(17) $$\begin{array}{c} {\hat{\Theta }}_{k}=\arg\min_{\Theta }-\sum_{s}\log p\left(Y_{s,k}|\Theta ,S_{s}\right), \end{array}$$

in which Θ contains the delays, gains, and angles of all the paths, as well as the number of paths. Since the number of paths is unknown and paths are not all resolvable, model order selection techniques can be applied, for example, by adding a regularizer to (17) ([11], Section 5.2), for example, following the minimum description length (MDL) or Akaike information criterion (AIC). The dense multipath can be treated as a stochastic process, by writing (14) as

(18) $$\begin{array}{c} Y_{s,k}=\sum_{i=0}^{I_{k}-1}g_{k}^{i,0}a_{R}\left(\theta_{k}^{i,0}\right)a_{T}^{H}\left(\phi_{k}^{i,0}\right)e^{-ȷ2\pi s\Delta f\tau_{k}^{i,0}}S_{s}+W_{s,k}^{\mathrm{dm}}+N_{s,k}, \end{array}$$

where $W_{s,k}^{\mathrm{dm}}$ is the dense multipath containing all the contributions from the diffuse paths. The dense multipath is then modeled as complex Gaussian process with zero mean and covariance $R_{k}^{\mathrm{dm}}$, possibly with unknown elements. Then the entire noise process has covariance $R_{k}^{\mathrm{dm}}+\sigma^{2}I_{M_{R}T}$. Alternatively, we can estimate the geometric parameters of the dominant diffuse multipath components from MIMO channel measurements, which leads to a multidimensional harmonic retrieval (HR) problem [48]. Numerous HR techniques have been developed, ranging from multidimensional searching or optimization based, such as multiple signal classification (MUSIC), ML and CS techniques [49], and polynomial-rooting or matrix-shifting based search-free methods, such as root-MUSIC [14] and estimation of parameters by rotational invariant techniques (ESPRIT) and their multidimensional extensions [50]. Due to its simplicity and high-resolution capability, ESPRIT-type algorithms have become one of the popular HR techniques [51].

### 4.2. ESPRIT Channel Estimator

For notational convenience, we drop the time index *k*. From (14), the received signal on subcarrier *s* is of the form

(19) $$\begin{array}{c} Y_{s}=H_{s}S_{s}+N_{s}, \end{array}$$

where $H_{s}$ is the channel frequency response, $S_{s}$ is a known pilot signal with orthogonality property ($S_{s}S_{s}^{H}=\beta I_{M_{T}\times M_{T}}$), and $N_{s}$ is i.i.d. Gaussian noise. Then we have

(20) $$\begin{array}{c} X_{s}=\frac{1}{\beta }Y_{s}S_{s}^{H}=H_{s}+\frac{1}{\beta }N_{s}S_{s}^{H}=H_{s}+W_{s}, \end{array}$$

where $W_{s}$ is also i.i.d. Gaussian noise with a scaled covariance matrix.

#### 4.2.1. Observations in Tensor Form

We utilize a Tensor framework to exploit the *R*-D grid structure inherent in the data, as well as the Vandermonde structure in angle and delay domains. To this end, we map from geometric channel parameters to spatial frequencies by

(21) $$\begin{array}{cc} \mu_{\left(1\right)}^{p} & =\pi \sin\left(\theta_{\mathrm{az}}^{p}\right)\cos\left(\theta_{\mathrm{el}}^{p}\right),\;\mu_{\left(2\right)}^{p}=\pi \sin\left(\theta_{\mathrm{el}}^{p}\right),\\ \mu_{\left(3\right)}^{p} & =\pi \sin\left(\phi_{\mathrm{az}}^{p}\right)\cos\left(\phi_{\mathrm{el}}^{p}\right),\;\mu_{\left(4\right)}^{p}=\pi \sin\left(\phi_{\mathrm{el}}^{p}\right),\\ \mu_{\left(5\right)}^{p} & =2\pi \Delta_{f}\tau^{p}. \end{array}$$

For subcarrier *i*, $X_{i}$ and $W_{i}$ are $M_{3}M_{4}\times M_{1}M_{2}$ matrices. We convert these $M_{5}$ matrices (one per subcarrier) to a 5D tensor of suitable dimension, X, H and $W\in C^{M_{1}\times M_{2}\times M_{3}\times M_{4}\times M_{5}}$. For the *p*-th path, the equivalent 5D array steering tensor can be written as

(22) $$A^{p}=a\left(\mu_{\left(1\right)}^{p}\right)\circ a\left(\mu_{\left(2\right)}^{p}\right)\circ \cdots \circ a\left(\mu_{\left(5\right)}^{p}\right),$$

where ∘ represents the outer product (Note that $A=a_{1}\circ a_{2}=a_{1}a_{2}^{T}$, and $A=a_{1}\circ a_{2}\circ a_{3}$ with $A_{ijk}=a_{1i}a_{2j}a_{3k}$.), $\mu_{\left(r\right)}^{p}$ is the spatial frequency of the *p*-th source in the *r*-th dimension, $a\left(\mu_{\left(r\right)}^{p}\right)\in C^{M_{r}\times 1}$ is equivalent to the uniform linear array steering vector composed of $M_{r}$ sensors, p=1,2,⋯,P, where *P* is the total number of paths, and r=1,2,⋯,5. This allows us to express the observation as

(23) $$X=\sum_{p=1}^{P}g^{p}A^{p}+W\in C^{M_{1}\times M_{2}\times \cdots \times M_{5}},$$

where $g^{p}$ denotes the complex path gain of the *p*-th path.

#### 4.2.2. Shift Invariance

We now introduce the multidimensional shift invariant structure in each dimension *r*, in order to apply Tensor-ESPRIT. We first introduce so-called *selection matrices* $J_{1,(r)},J_{2,(r)}\in R^{M_{r}^{\left(\mathrm{sel}\;\right)}\times M_{r}}$, which select $M_{r}^{\left(\mathrm{sel}\;\right)}$ out of $M_{r}$ elements belonging to the first and the second subarray in the *r*-th mode, respectively. For example, when $M_{r}^{\left(\mathrm{sel}\;\right)}=M_{r}-1$, we have

(24) $$J_{1,(r)}=\left[\begin{array}{cc} I_{M_{r}-1} & 0_{\left(M_{r}-1\right)\times 1} \end{array}\right]\;\mathrm{and}\;J_{2,(r)}=\left[\begin{array}{cc} 0_{\left(M_{r}-1\right)\times 1} & I_{M_{r}-1} \end{array}\right],$$

where I and 0 denote the identity matrix and zero vector, respectively, and the sizes are defined in subscripts. Secondly, we introduce $A_{\left(r\right)}=\left[\begin{array}{cccc} a\left(\mu_{\left(r\right)}^{1}\right) & a\left(\mu_{\left(r\right)}^{2}\right) & \cdots & a\left(\mu_{\left(r\right)}^{P}\right) \end{array}\right]$, which has the shift invariance property

(25) $$J_{1,(r)}A_{\left(r\right)}\Phi_{\left(r\right)}=J_{2,(r)}A_{\left(r\right)},$$

where $\Phi_{\left(r\right)}=\mathrm{diag}\left(e^{ȷ\cdot \mu_{\left(r\right)}^{1}},\;e^{ȷ\cdot \mu_{\left(r\right)}^{2}},\;\ldots ,\;e^{ȷ\cdot \mu_{\left(r\right)}^{L}}\right)$ is a diagonal matrix that contains the unknown parameters $\mu_{l}^{\left(r\right)},l=1,2,\cdots ,L$, in dimension *r*.

#### 4.2.3. Tensor-ESPRIT

In order to obtain the subspace spanned by $A_{\left(r\right)}$, we take CANDECOMP/PARAFAC (CP) decomposition on X [52]. Because the total number of paths *P* is unknown, model order selection techniques [53] can be utilized to estimate $\hat{P}$. In general, the estimated $\hat{P}\ll P$ for rough surfaces with hundreds of closely located diffuse paths.

(26) $$X=\sum_{p=1}^{\hat{P}}{\hat{g}}^{p}u_{\left(1\right)}^{p}\circ u_{\left(2\right)}^{p}\circ \cdots \circ u_{\left(5\right)}^{p}\equiv \left[\begin{array}{c} \hat{g};\;U_{\left(1\right)},\;U_{\left(2\right)},\;\cdots ,\;U_{\left(5\right)} \end{array}\right],$$

where $\hat{g}=\left[{\hat{g}}^{1}\;\;{\hat{g}}^{2}\;\;\cdots \;\;{\hat{g}}^{\hat{P}}\right]$ are the estimated path gains, and $U_{\left(r\right)}=\left[u_{\left(r\right)}^{1}\;u_{\left(r\right)}^{2}\;\cdots \;u_{\left(r\right)}^{\hat{P}}\right]$ is the estimated signal subspace with normalized column vectors $u_{\left(r\right)}^{p}$. Note that after taking CP decomposition, the path gains $g^{p}$ and channel parameter associations are achieved synchronously. In other words, $g^{p}$ corresponds to a unique $u_{\left(r\right)}^{p}$ in each dimension *r*, so that the output of (26) is $\left\{{\hat{g}}^{p},u_{\left(1\right)}^{p},u_{\left(2\right)}^{p},\cdots ,u_{\left(5\right)}^{p}\right\},p=1,2,\cdots ,\hat{P}$.

Since $A_{\left(r\right)}$ and $U_{\left(r\right)}$ span the same subspace, $A_{\left(r\right)}=U_{\left(r\right)}T_{\left(r\right)}$, where $T_{\left(r\right)}\in C^{\hat{P}\times \hat{P}}$ is a non-singular transform matrix. Entering this relation in (25), we obtain

(27) $$J_{1,(r)}U_{\left(r\right)}\Psi_{\left(r\right)}=J_{2,(r)}U_{\left(r\right)},$$

where $\Psi_{\left(r\right)}={\left(T_{\left(r\right)}\right)}^{-1}\Phi_{\left(r\right)}T_{\left(r\right)}$. In (27), only $\Psi_{\left(r\right)}$ is unknown, but can be estimated via the least squares method:

(28) $$\begin{array}{c} {\hat{\Psi }}_{\left(r\right)}={\left(J_{1,(r)}U_{\left(r\right)}\right)}^{†}J_{2,(r)}U_{\left(r\right)}, \end{array}$$

where $\cdot^{†}$ denotes the pseudo-inverse. Since the matrix $\Psi_{\left(r\right)}$ is similar to the diagonal matrix $\Phi_{\left(r\right)}$, they share the same eigenvalues. Hence, the spatial frequency can be recovered as ${\hat{\mu }}_{\left(r\right)}^{p}=\arg\left(\lambda_{\left(r\right)}^{p}\right)$, where $\lambda_{\left(r\right)}=\left[\lambda_{\left(r\right)}^{1},\lambda_{\left(r\right)}^{2},\cdots ,\lambda_{\left(r\right)}^{\hat{P}}\right]$ are the eigenvalues of $\Psi_{\left(r\right)}$. The $\{{\hat{\mu }}_{\left(r\right)}^{p}\},r=1,2,\cdots ,5$, map to delay ${\hat{\tau }}^{p}$, AOA pair ${\hat{\theta }}^{p}={\left[{\hat{\theta }}_{\mathrm{az}}^{p},\;{\hat{\theta }}_{\mathrm{el}}^{p}\right]}^{T}$, and AOD pair ${\hat{\phi }}^{p}={\left[{\hat{\phi }}_{\mathrm{az}}^{p},\;{\hat{\phi }}_{\mathrm{el}}^{p}\right]}^{T}$ via (21).

Finally, each tuple $\left[{\hat{\tau }}^{p},\;{\hat{\theta }}_{\mathrm{az}}^{p},\;{\hat{\theta }}_{\mathrm{el}}^{p},\;{\hat{\phi }}_{\mathrm{az}}^{p},\;{\hat{\phi }}_{\mathrm{el}}^{p},\;{\hat{g}}^{p}\right]$, for $p\in \{1,\ldots ,\hat{P}\}$ is returned as the output of the channel estimator. We denote this combined output as Z.

The most computationally demanding part of channel parameter estimation is the CP decomposition. In (Reference [54], Table 1), the complexities of major computations in popular CP decomposition algorithms is summarized. For example, the alternating least squares (ALS) algorithm with line search has a complexity of order $O\left(2^{R}\hat{P}J+R{\hat{P}}^{3}\right)$, where $J=\prod_{r=1}^{R}M_{r}$ and R=5 and $\hat{P}$ denotes the total number of paths.

## 5. Channel Parameter Clustering

At time step *k*, the channel estimator provides a set of channel parameter estimates $Z_{k}$, $Z_{k}=\left\{z_{k}^{0},z_{k}^{1},\cdots ,z_{k}^{{\hat{P}}_{k}-1}\right\}$, and we need to cluster $Z_{k}$ based on different landmarks. However, clustering is challenging, since $Z_{k}$ is in a 6D space, and the delay, angle, and gain are in different scales and spaces, so that they should be properly weighted. We also do not have any advance knowledge of the number of landmarks in the environment. In this section, we try to address these challenges. For brevity, we will omit the time index *k*.

### 5.1. Background

As a general term, a *cluster* is defined as a collection of objects that are similar to each other in some agreed-upon sense [55]. In radio channel analysis, a cluster is usually described as a group of multipath components (MPCs) with the same parameter distribution. The MPCs that have similar values in delay and angular domain are jointly classified as a single cluster, which reflects the physical environment, as similar MPCs are usually spatially close to each other, for example, reach the receiver via the same landmark.

Clustering methodology in radio channel analysis has been expanded from visual clustering to automatic clustering, where the visual clustering is usually valid for data with limited dimensions [55,56], while the automatic clustering can handle data with more than three dimensions [57]. Automatic clustering algorithms, focusing on the parameter space of MPCs, such as Hierarchical, K-means, and Gaussian mixture, have been widely used in radio channel characterizations [32,58,59,60,61]. Hierarchical clustering algorithms cluster the data based on a binary cluster tree, which is limited by the data size, and therefore limited to small data sets. K-means [62] assumes a known number of clusters and iteratively assigns data to each cluster and computes cluster centroids until the cluster centroids are converged. While K-means is a widely used clustering method, it suffers from several drawbacks: (i) the number of clusters is predefined, which limits the capabilities to reflect the reality of the environment; (ii) it cannot cope with outliers, which implies all the observed data will eventually be part of some clusters, even observations that are far away in 6D space and should be considered as outliers; (iii) it usually gives spherical-like shape clusters. MPCs that reflect other physical properties can be assigned as the edge of a spherical cluster, which has notable effects on the cluster properties, that is, cluster centroids, and loses the ability to link to physical reality. The more sophisticated affinity propagation [63] partially avoids these problems, but still leads to circular clusters and has very high complexity. Gaussian mixture clustering [57] gives more variation to the shapes of the clusters extracted with K-means, and follows the similar structure of K-means. It can give clusters in different shape if the original data is not distributed circularly. However, the method is complex and converges slowly.

Density-based spatial clustering of applications with noise (DBSCAN) [64] is one of the most widely applied clustering methods for data sorting. Without a predefined number of clusters, DBSCAN utilizes two critical parameters—the minimum number of points clustered together for a region to be considered dense, and a distance measure to locate the points in the neighborhood of any point. The density-based clustering method has no limitations on the number of clusters, uses a dense distance, and gives more freedom regarding the shape of clusters. While we have evaluated different methods in Section 7, here we only present the best performing method, which is an extension of the DBSCAN algorithm.

### 5.2. Modified DBSCAN

In this section, we will present the DBSCAN clustering method with the obtained channel estimates. The method involves three phases—(i) the mapping of the geometric channel parameters (delay and angles) to a 3D point; (ii) clustering with DBSCAN; (iii) refinement by using the channel gain.

#### 5.2.1. Phase 1: 5D to 3D Mapping

Distinct from existing work where the channel parameters are used as features for clustering [28,61], we transform the 5D channel parameter into a 3D position. In the absence of noise, this point should be on the surface of a landmark for NLOS paths, while in the presence of noise, the most likely estimation of the point can be founded close to the surface. The transformation reduces the dimensionality, and avoids different scales and spaces problems. Hence, the 5D parameters, that is, the delay ${\hat{\tau }}^{p}$, the AOA pair ${\hat{\theta }}^{p}={\left[{\hat{\theta }}_{\mathrm{az}}^{p},\;{\hat{\theta }}_{\mathrm{el}}^{p}\right]}^{T}$ and the AOD pair ${\hat{\phi }}^{p}={\left[{\hat{\phi }}_{\mathrm{az}}^{p},\;{\hat{\phi }}_{\mathrm{el}}^{p}\right]}^{T}$ can be converted to a point ${\hat{x}}^{p}$.

**Figure 4:**
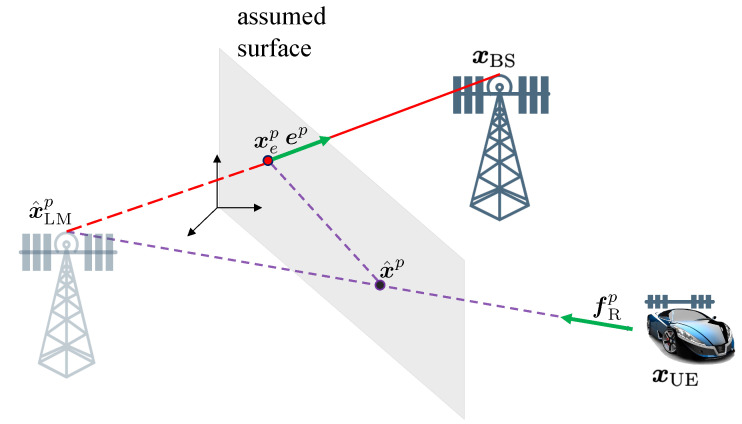
Illustration of the 3D transformation from the 5D parameters: from the channel estimates, a hypothesized landmark (a virtual anchor) location ${\hat{x}}_{\mathrm{LM}}^{p}$ is determined. From the landmark and BS locations, the incidence point ${\hat{x}}^{p}$ is derived.

The general idea to transform the 5D parameters into a 3D point is illustrated in Figure 4. Specifically, for the *p*-th diffuse multipath, a virtual anchor position can be estimated as

(29) $$\begin{array}{c} {\hat{x}}_{\mathrm{LM}}^{p}=x_{\mathrm{UE}}+c\left({\hat{\tau }}^{p}-\hat{B}\right)f_{R}^{p}, \end{array}$$

where *c* is the speed of light, $\hat{B}$ is the estimated clock bias, $f_{R}^{p}$ is the unit vector pointing along with the direction of arrival, given by

(30) $$\begin{array}{c} f_{R}^{p}=\left[\begin{array}{c} \cos{\hat{\theta }}_{\mathrm{az}}^{p}\cos{\hat{\theta }}_{\mathrm{el}}^{p}\\ \sin{\hat{\theta }}_{\mathrm{az}}^{p}\cos{\hat{\theta }}_{\mathrm{el}}^{p}\\ \sin{\hat{\theta }}_{\mathrm{el}}^{p} \end{array}\right]. \end{array}$$

Given ${\hat{x}}_{\mathrm{LM}}^{p}$ and $x_{\mathrm{BS}}$, a hypothesized surface can be defined via a point on the surface $x_{e}^{p}=\left(x_{\mathrm{BS}}+{\hat{x}}_{\mathrm{LM}}^{p}\right)/2$ and the normal vector to the surface $e^{p}=\left(x_{\mathrm{BS}}-{\hat{x}}_{\mathrm{LM}}^{p}\right)/∥x_{\mathrm{BS}}-{\hat{x}}_{\mathrm{LM}}^{p}∥$. With knowledge of ${\hat{x}}_{\mathrm{LM}}^{p}$ and the surface, the incidence point location can be computed from from Snell’s law of reflection as

(31) $$\begin{array}{c} {\hat{x}}^{p}={\hat{x}}_{\mathrm{LM}}^{p}+\frac{{\left(x_{e}^{p}-{\hat{x}}_{\mathrm{LM}}^{p}\right)}^{T}e^{p}}{{\left(x_{\mathrm{UE}}-{\hat{x}}_{\mathrm{LM}}^{p}\right)}^{T}e^{p}}\left(x_{\mathrm{UE}}-{\hat{x}}_{\mathrm{LM}}^{p}\right). \end{array}$$

Note that this method also works for the LOS path, and it will return a point near ${\hat{x}}_{\mathrm{BS}}$, since ${\hat{x}}_{\mathrm{LM}}^{p}$ and $x_{e}^{p}$ are both approximate to ${\hat{x}}_{\mathrm{BS}}$. From Z, we obtain the corresponding $\hat{P}$ 3D points $D=\{{\hat{x}}^{0},{\hat{x}}^{1},\cdots ,{\hat{x}}^{{\hat{P}}_{k}-1}\}$. Next, we will perform the clustering methods with the 3D positions of points.

#### 5.2.2. Phase 2: Clustering with DBSCAN

In DBSCAN [64], all points are classified into either clusters or identified as noise. In a specific cluster C, the *core points* have an ϵ-*neighbourhood* with $N_{\min}\in N$ points. We define a distance measure $d:R^{3}\times R^{3}\to R$ from which the ϵ–*neighbourhood* of the *p*-th point is defined as

(32) $$\begin{array}{c} C_{p}\overset{\Delta }{=}\left\{{\hat{x}}^{q}\in D:d\left({\hat{x}}^{p},{\hat{x}}^{q}\right)<\epsilon \right\}. \end{array}$$

In this paper, we use $d\left({\hat{x}}^{p},{\hat{x}}^{q}\right)=∥{\hat{x}}^{p}-{\hat{x}}^{q}∥$. The main idea of DBSCAN is that clusters are not characterized by their variance (as in K-means), but by *density-reachability*. In particular, ${\hat{x}}^{q}$ is directly density-reachable from ${\hat{x}}^{p}$, if and only if $|C_{p}|\geq N_{\min}$ and ${\hat{x}}^{q}\in C_{p}$. In addition, ${\hat{x}}^{p}$ is density-reachable from ${\hat{x}}^{q}$, if there exists an ordered sequence of points ${\hat{x}}^{p_{0}}={\hat{x}}^{q},{\hat{x}}^{p_{1}},\ldots ,{\hat{x}}^{p_{n}}={\hat{x}}^{p}$ in D, where ${\hat{x}}^{p_{i+1}}$ is directly density-reachable from ${\hat{x}}^{p_{i}}$. If the sphere of ${\hat{x}}^{p}$ contains at least $N_{\min}$ points, that is, $\left|C_{p}\right|\geq N_{\min}$, the point ${\hat{x}}^{p}$ will be a core point of a cluster. The border points can have a smaller size ϵ-*neighbourhood*, but can be density-reachable from the core points.

Given the parameters ϵ and $N_{\min}$, we can discover a cluster in a two-step approach: (i) choose an arbitrary point from the database satisfying the core point conditions as a seed; (ii) find all points that are density-reachable from the seeds [64]. We follow these two steps, that is, to identify a core point, to find a cluster with the core point, and to find all clusters. The algorithms are provided in Algorithm 1. Note that Algorithm  2 is used in Algorithm  1 to find all points in each cluster. The output of the clustering is a partitioning of Z (or D) into clusters $P_{l}$ and a set of noise points $P_{N}$.
**Algorithm 1:** DBSCAN for Clustering**Input:** Points $\left\{{\hat{x}}^{0},{\hat{x}}^{1},\cdots ,{\hat{x}}^{{\hat{P}}_{k}-1}\right\}$, threshold ϵ, and $N_{\min}$;**Output:** All clusters and the associated points.1:Compute pair-wise Euclidean distance for all points, store them in D, and label all points unvisited;2:l←0 and p←0; // cluster index *l* and point index *p*3:**while**p<P**do**4:    **if** Point *p* is unvisited **then**5:        Label point *p* as visited;6:        Find all neighbours within ϵ distance of *p*;7:        **if** ϵ-*neighbourhood* is less than $N_{\min}$ **then**8:           Classify the point *p* as noise;9:        **else**10:           l←l+1; // *new cluster*11:           Discover all points in cluster *l* by using **Algorithm** 2.12:        **end if**13:    **end if**14:**end while**


**Algorithm 2:** Find All Points in Cluster *l***Input:** Cluster index *l*, point index *p* and its ϵ-*neighbourhood*
$C_{p}$;**Output:** The associated points in cluster *l*.
1:Classify *p* into cluster *l*;2:Store all indexes of the points in $C_{p}$ in vector c;3:k←1;4:
**while** $k\leq \left|c\right|$ **do**
5:    $j\leftarrow c_{k}$;6:    **if** Point ${\hat{x}}^{j}$ is unvisited **then**7:        Label point ${\hat{x}}^{j}$ as visited;8:        Find all neighbours within ϵ distance of ${\hat{x}}^{j}$, that is, $C_{j}$;9:        **if** $\left|C_{j}\right|\geq N_{\min}$ **then**10:           Add all indexes in $C_{j}$ to c;11:        **end if**12:    **end if**13:    **if** ${\hat{x}}^{j}$ is not classified to any cluster **then**14:        Classify ${\hat{x}}^{j}$ in cluster *l*;15:    **end if**16:    k←k+1;17:
**end while**



#### 5.2.3. Phase 3: Extract Isolated Specular Paths and Outliers Using Channel Gain

The DBSCAN clustering has two drawbacks in our application:

- The tensor ESPRIT channel estimator from Section 4 can generate estimates, whose 3D points (as obtained in Section 5.2.1) are still on or near the corresponding surfaces, but are far away from the cluster centers. Hence, they are informative for the SLAM algorithm, but are part of $P_{N}$, so they are not clustered correctly. We have observed that the channel gains of these paths are very small.
- The LOS path and specular paths from smooth surfaces are not part of any cluster, as such landmarks have one or few associated paths. We have observed that the channel gains of these paths are very large (approximately following the path loss models from Section 2.3).

To solve these problems, we use the estimated channel gains. We partition $P_{N}$ into two sets according to the channel gains.

(33) $$\begin{array}{cc} P_{N,L} & =\{q:c\left({\hat{\tau }}^{p}-\hat{B}\right)|{\hat{g}}^{p}|\frac{4\pi }{\lambda }\leq \delta \} \end{array}$$

(34) $$\begin{array}{cc} P_{N,H} & =\{q:c\left({\hat{\tau }}^{p}-\hat{B}\right)|{\hat{g}}^{p}|\frac{4\pi }{\lambda }>\delta \}, \end{array}$$

where δ is a predefined threshold, $\hat{B}$ is the estimated clock bias, and *c* is the speed of light. The scaling with $c\left({\hat{\tau }}^{p}-\hat{B}\right)\frac{4\pi }{\lambda }$ is added to compensate for the path loss. For each of the high-gain paths in $P_{N,H}$, we create a new cluster. For each of the low-gain paths in $p\in P_{N,L}$, we find the nearest cluster $l^{*}$ as

(35) $$\begin{array}{c} l^{*}=\arg\min_{l}\min_{q\in P_{l}}d\left({\hat{x}}^{p},{\hat{x}}^{q}\right). \end{array}$$

and add *p* to cluster $P_{l^{*}}$.

The overall computations in channel parameter clustering consist of three parts, that is, the computations in each phase. Given the number of paths $\hat{P}$, the transition from 5D to 3D mapping requires $O\left(\hat{P}\right)$ computations. According to Reference [64], the complexity of the DBSCAN algorithm without the use of index structure for acceleration is $O\left({\hat{P}}^{2}\right)$. The last phase requires $O\left(\hat{P}\right)$ computations. Therefore, the overall complexity is $O\left({\hat{P}}^{2}\right)$.

## 6. Likelihood Function for SLAM

After clustering, the measurements $Z_{k}$ are grouped into clusters $Z_{k}=\left\{Z_{k}^{0},Z_{k}^{1},\ldots ,Z_{k}^{{\hat{I}}_{k}-1}\right\}$, using Algorithm 1, where ${\hat{I}}_{k}$ is the number of estimated clusters. The clustered measurements will be the input into the SLAM filter during the update process, via an appropriate likelihood function. For brevity, we omit the time index *k* again. We work with the compact representation of the landmark state from Section 2.2. Hence, our goal is to determine $\ell (Z^{i}|x_{\mathrm{LM}},s,m)$, where $x_{\mathrm{LM}}$ is the landmark position (i.e., the BS location or a virtual anchor location) and m∈{BS,SM,MR,VR} is the landmark state. This likelihood function will influence the probabilities of different associations between landmarks and clusters, and it will also influence the particle weight and the distribution of the landmark given a state s and the association.

### 6.1. Background

In previous 5G SLAM works, Reference [21] assumed there is at most one measurement from each source; the channel gain is not used, and the inter-path interference is not considered. The measurements are only generated by known multivariate Gaussian distributions, then the likelihood function falls into the likelihood of the signal measurements, which follows the Gaussian format. According to the PMBM formalism, multiple measurements per source should follow a Poisson or Bernoulli distribution. Under the standard point model, the likelihood function corresponds to a Bernoulli distribution, with [65]

(36) $$\begin{array}{c} \ell \left(Z^{i}|x_{\mathrm{LM}},s,m\right)=\left\{\begin{array}{cc} 1-p_{D} & Z^{i}=\emptyset \\ p_{D}p\left(z^{i,0}|x_{\mathrm{LM}},s,m\right) & Z^{i}=\left\{z^{i,0}\right\}\\ 0 & \mathrm{else}, \end{array}\right. \end{array}$$

where $p_{D}$ is the detection probability. Under the extended target model, the likelihood function corresponds to a Poisson point process (PPP) ([25], Equation (5)):

(37) $$\begin{array}{c} \ell \left(Z^{i}|x_{\mathrm{LM}},s,m\right)=e^{-\gamma_{m}}\gamma_{m}^{|Z^{i}|}\prod_{l=0}^{|Z^{i}|-1}p\left(z^{i,l}|x_{\mathrm{LM}},s,m\right), \end{array}$$

where $\gamma_{m}\geq 0$ is the Poisson rate for surfaces of type *m*. For both (36) and (37), it has been proven that the PMBM density is a conjugate prior.

### 6.2. Likelihood Function

In this section, we describe the proposed likelihood function. We here assume that the measurements within each cluster $Z^{i}$ are independent, as diffuse points are generated independently. We also assume the number of paths $|Z^{i}|$ only depends on the source type *m*. It is important to note that for measurements from diffuse paths, a measurement is a function of a *random incidence point on the surface*. In order to express the measurements as a function of the compact state, we propose the following approach.

We first separate $Z^{i}$ into two parts, the path with the shortest delay $\left\{z^{i,0}\right\}$, which is the closest path to the specular component, and any remaining paths ${\tilde{Z}}^{i}=\left\{z^{i,1},z^{i,2},\cdots ,z^{i,|Z^{i}|-1}\right\}$, which we view as diffuse multipath. We associate the deterministic incidence point $x^{i,0}$ with the assumed specular component, which can be derived by (31) using $x_{\mathrm{BS}}$ and $x_{\mathrm{LM}}$. Therefore, we can write $p\left(z^{i,0}|x_{\mathrm{LM}},s,m\right)=p\left(z^{i,0}|x^{i,0},s,m\right)$, for $z^{i,0}$, which is in the desired form (16). Hence

(38) $$\begin{array}{c} \ell \left(Z^{i}|x_{\mathrm{LM}},s,m\right)=\left\{\begin{array}{cc} 1-p_{D} & Z^{i}=\emptyset \\ p_{D}p\left(z^{i,0}|x_{\mathrm{LM}},s,m\right)\ell \left({\tilde{Z}}^{i}|x_{\mathrm{LM}},s,m\right) & \mathrm{else}. \end{array}\right. \end{array}$$

In other words, the object may be miss-detected with probability $1-p_{D}$. If the object is detected, then the likelihood contains a contribution from the first arriving path (the specular path, which is directly related to the VA location or BS location) and from the set of all remaining paths.

Note that as a special case for m=BS there can be at most one associated measurement, so that

(39) $$\begin{array}{c} \ell \left(Z^{i}|x_{\mathrm{LM}},s,m=\mathrm{BS}\right)=\left\{\begin{array}{cc} 1-p_{D} & Z^{i}=\emptyset \\ p_{D}p\left(z^{i,0}|x_{\mathrm{LM}},s,m\right) & Z^{i}=\left\{z^{i,0}\right\}\\ ,0 & \mathrm{else}. \end{array}\right. \end{array}$$

which is in the desired form. Hence, we can focus on the diffuse paths. As the measurements contain both a specular component and diffuse paths, the likelihood function does not follow the standard models (36)–(37). Nevertheless, we use the likelihood in the PMBM filter without any proof of conjugacy or optimality.

#### 6.2.1. Likelihood for Diffuse Paths

We recall from Section 2.3 that diffuse multipath originates from random points $x^{i,l}$ on the surface and from Section 4 that the estimates points will differ from those from Section 2.3, due to the finite resolution of the receiver. Hence, it is *fundamentally impossible* to estimate the original random incidence points $x^{i,l}$ from the observations ${\tilde{Z}}^{i}$. To avoid estimating $x^{i,l}$, we propose estimating an *artificial incidence point*${\tilde{x}}^{i,l}$ from $z^{i,l>0}$ as the projection of ${\hat{x}}^{i,l}$ (obtained using the method in Section 5.2.1) onto the surface. The projection point ${\tilde{x}}^{i,l}$ can be derived by

(40) $$\begin{array}{c} {\tilde{x}}^{i,l}={\hat{x}}^{i,l}+{\left(x_{e}-{\hat{x}}^{i,l}\right)}^{T}e\;e, \end{array}$$

where $x_{e}=\left(x_{\mathrm{BS}}+x_{\mathrm{LM}}\right)/2$ is a point on the surface, and $e=\left(x_{\mathrm{BS}}-x_{\mathrm{LM}}\right)/∥x_{\mathrm{BS}}-x_{\mathrm{LM}}∥$ is a normal to the surface. Then, $p(z^{i,l}|x_{\mathrm{LM}},s,m)$ can be expressed in the desired form $p(z^{i,l}|{\tilde{x}}^{i,l},s,m)$, where ${\tilde{x}}^{i,l}$ is the artificial incidence point that gave rise to measurement $z^{i,l}$. The likelihood can be further simplified by noticing the error component of ${\tilde{x}}^{i,l}$, which is only due to the the projection distance of ${\hat{x}}^{i,l}$ to the surface. We therefore use this distance directly as a compressed measurement to replace the delay and angles

(41) $$\begin{array}{c} {\hat{d}}^{i,l}\left(z^{i,l}\right)=e^{T}\left(x_{e}-{\hat{x}}^{i,l}\right). \end{array}$$

**Figure 5:**
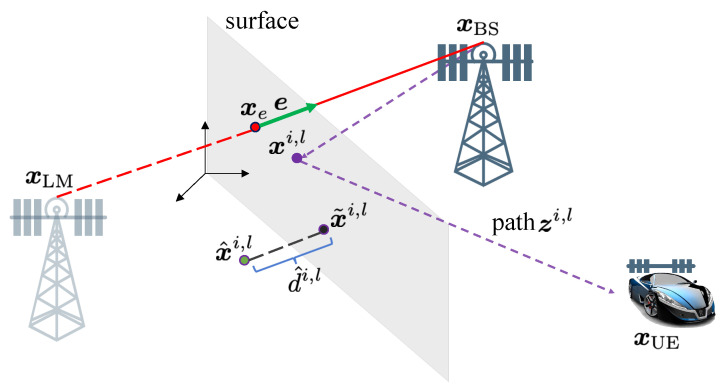
The principle of finding ${\tilde{x}}^{i,l}$ and calculating ${\hat{d}}^{i,l}$ using $z^{i,l}$, $x_{\mathrm{LM}}$ and $x_{\mathrm{BS}}$.

Figure 5 shows the principle of calculating ${\hat{d}}^{i,l}\left(z^{i,l}\right)$.

We thus have a general model for the likelihood function associated with the diffuse paths, where the cardinality distribution is arbitrary and (cf. (Reference [18], Equation (1))

(42) $$\begin{array}{cc} \; & \ell \left({\tilde{Z}}^{i}|x_{\mathrm{LM}},s,m\right)=p(|{\tilde{Z}}^{i}\left||m)\right|{\tilde{Z}}^{i}|!\prod_{l=1}^{|{\tilde{Z}}^{i}|}p\left(\left[{\hat{d}}^{i,l}\left(z^{i,l}\right),{\hat{g}}^{i,l}\right]|x_{\mathrm{LM}},s,m\right), \end{array}$$

in which all constituent distributions can be obtained from the simulation of a channel estimator or provided directly in closed-form by a channel estimator. In (42), the first term $p(|{\tilde{Z}}^{i}||m)$ represents the cardinality distribution, $|{\tilde{Z}}^{i}|!$ is a normalization constant to ensure that $\ell ({\tilde{Z}}^{i}|x_{\mathrm{LM}},s,m)$ integrates to one over ${\tilde{Z}}^{i}$. The element in ${\tilde{Z}}^{i}$ are represented in compressed format through $\left[{\hat{d}}^{i,l}\left(z^{i,l}\right),{\hat{g}}^{i,l}\right]$ and are modeled as independent and identically distributed.

#### 6.2.2. Clustering Errors and Marginal Likelihood Function

Due to clustering errors and channel estimator errors, it is possible that the specular path is not associated with the set of the specular paths. Then the shortest path in $Z^{i}$ may be a diffuse path, leading to low likelihood $p(z^{i,0}|x_{\mathrm{LM}},s,m)$, so that the SLAM method is likely to view the source of this measurement cluster as a new landmark, which is not desirable. In order to solve this problem, we introduce $p_{m}\in \left(0,1\right]$, which is the probability that the first path in a cluster is in fact the specular path. This probability can be determined by observation of the overall clustering performance. By marginalizing over the event of missing the specular path in a cluster, the likelihood function can be written as

(43) $$\begin{array}{cc} p(z^{i,0}|x_{\mathrm{LM}},s,m) & =p_{m}p\left(z^{i,0}|x_{\mathrm{LM}},s,m\right)\\ \; & +\left(1-p_{m}\right)\frac{p(|{\tilde{Z}}^{i}\left|+1|m)(\right|{\tilde{Z}}^{i}\left|+1\right)}{p(|{\tilde{Z}}^{i}||m)}p\left(\left[{\hat{d}}^{i,0}\left(z^{i,0}\right),{\hat{g}}_{k}^{i,0}\right]|x_{\mathrm{LM}},s,m\right), \end{array}$$

where the factor $p(|{\tilde{Z}}^{i}\left|+1|m)(\right|{\tilde{Z}}^{i}\left|+1)/p(\right|{\tilde{Z}}^{i}\left||m\right)$ is due to there being an additional specular path, which was not present in the cluster. The complexity of the likelihood function is $O\left(|{\tilde{Z}}^{i}|\right)$, as we consider $\left(|{\tilde{Z}}^{i}|+1\right)$ paths.

## 7. Results

In the implementation, all the codes are written in MATLAB, and the simulations and experiments are run on a MacBook Pro (15-inch, 2019) with a 2.6 GHz 6-Core Intel Core i7 processor and 16 Gb memory.

### 7.1. Simulation Parameters

We consider a scenario with a single vehicle with the initial state ${\left[70.7285\;m,0\;m,0\;m,\pi /2\;\mathrm{rad},22.22\;m/s,\pi /10\;\mathrm{rad}/s,1\;\mu s\right]}^{T}$, a BS located at ${\left[0\;m,0\;m,40\;m\right]}^{T}$, a SM, with the VA at ${\left[200\;m,0\;m,40\;m\right]}^{T}$, two MRs, with VAs at ${\left[0\;m,\pm 200\;m,40\;m\right]}^{T}$ and a VR, with the VA at ${\left[0\;m,-200\;m,40\;m\right]}^{T}$. The BS and the receiver are both equipped with a URA with 8×8 antennas. The vehicle moves around the BS with a known constant turn rate. Every time step, the BS sends 10×64 OFDM symbols to the vehicle with 200 subcarriers using the transmit power of 5.05 W at carrier frequency of 28 GHz. The subcarrier spacing is 0.5 MHz. The transmitted signals are disturbed by the noise with power spectral density of $4.0049\times 10^{-9}$ mW/Hz. The transition function of the movement, the initial vehicle state, the landmark locations, the process noise, the initial prior, the survival probability, the detection probability, the birth rate, the clutter intensity, and pruning thresholds are the same as in Reference [23].

### 7.2. Channel Estimation Results

**Figure 6:**
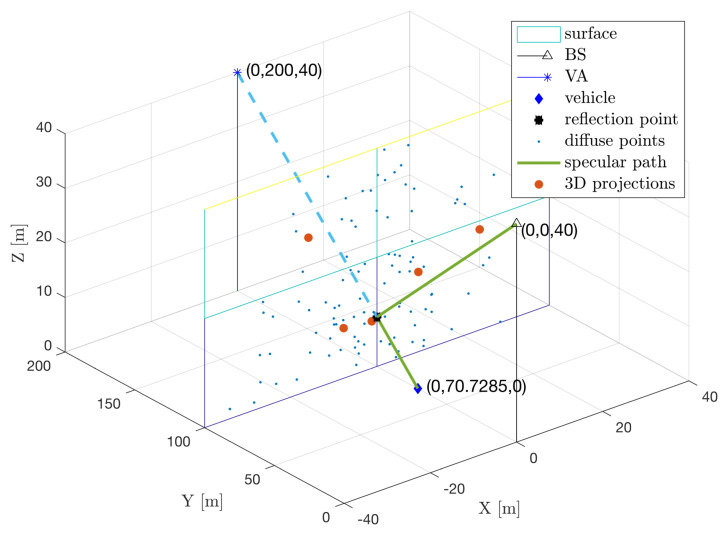
The 3D projection of channel estimation results of 100 diffuse path and a specular path from an MR.

In this section, we show the results of the tensor ESPRIT channel estimator for a MR surface, which generated one specular path and 100 diffuse paths. To visualize the channel estimator output, we transform the estimated TOAs, AOAs, AODs into 3D points. The result is shown in Figure 6, where the original diffuse points are marked with blue dots and the estimated diffuse points with red discs. We observe that the channel estimator returns only 5 paths, much less than the original 101 paths, due to the finite resolution of the receiver. All projected points are close to the surface and there is a projected point very close to the deterministic reflection point. This is because the specular path of the MR has larger power than the other diffuse paths, so it is less affected by inter-path interference.

### 7.3. Clustering Performance Evaluation

To evaluate the clustering performance, we generate 40 snapshots of the channel with known landmarks. We then obtain the channel estimates with 5D geometric parameters, and transform them into 3D point positions. The DBSCAN clustering algorithm is applied to each snapshot, with ϵ=30 m and $N_{\min}=2$. Then we use the estimated channel gain to modify the results, with δ=0.25. As a comparison, we also compare with other clustering methods, including the well-known K-means, gap statistics (‘GS’) [66], and affinity propagation (’AP’) [63] on the same data set. For the K-means, we assume that the number of clusters, *K*, is known, while for the remaining methods this priori knowledge is not needed.

To evaluate the clustering performance, we consider the following performance metrics, which use as ground-truth hand-labeled data: the clustering accuracy (CA), which is the percentage of correctly clustered points, and the impurity (IMP), which is the percentage of points clustered into a wrong cluster

(44) $$\begin{array}{cc} \mathrm{CA}(G,C) & =\frac{\max_{\gamma \in \Gamma }\left(\hat{P}+\sum_{(i,j)\in \gamma }\left(\right|G_{i}\cap C_{j}\left|-\right|C_{j}\left|\right)\right)}{\hat{P}} \end{array}$$

(45) $$\begin{array}{cc} \mathrm{IMP}(G,C) & =\frac{\sum_{k\in \{1,\cdots ,\hat{N}\}}\left(\right|C_{k}|-\max_{m\in \{1,\cdots ,N\}}|G_{m}\cap C_{k}\left|\right)}{\hat{P}}, \end{array}$$

where Γ is the set of all possible assignments; $G=\{G_{1},\cdots ,G_{N}\}$ is the ground truth clusters; $C=\{C_{1},\cdots ,C_{\hat{N}}\}$ is the cluster results provided by the clustering algorithm; $\hat{P}$ is the number of all target points. The performance is presented in Table 2. The proposed clustering method based on DBSCAN provides the best clustering performance, with an average clustering accuracy over 99%. The modified DBSCAN goes through all noise points using channel gain and refines the results, so it performs slightly better. K-means achieves an average accuracy of 94.63%. GS-based clustering method has an average accuracy of only 68.64% since the estimated value of the number of clusters can be erroneous. The AP clustering method has an average accuracy of 84.35% since it still based on space partition and the estimated value of the number of clusters can be erroneous either. In terms of impurity, the last three algorithms cluster measurements from different sources together when errors occur, but for DBSCANs this never happens, so that all points in each cluster correspond to one landmark. By observing the clustering results of modified DBSCAN, losing the shortest path from the same landmark happens rarely, and we have estimated $p_{m}\approx 1/200$.

**Figure 7:**
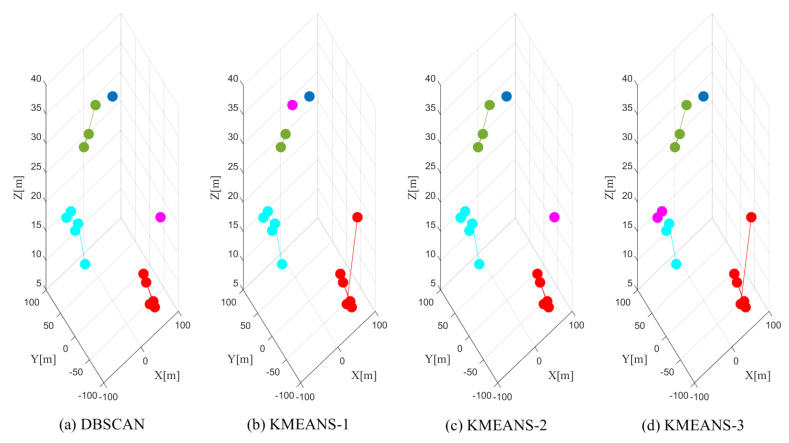
The clustering performance of DBSCAN and K-means.

Though the K-means algorithm shows good average clustering performance, it suffers from a few limitations as indicated in Section 5.1. This can be demonstrated in Figure 7, where we present the clustering results with DBSCAN and K-means with the data from the 33rd snapshot. We use different colors to indicate the different clusters obtained from the corresponding clustering methods. The proposed DBSCAN can correctly cluster the measurements into the right clusters shown in Figure 7a; however, K-means shows unstable clustering performance indicated in Figure 7b–d. This is because the clustering performance of K-means is highly dependent on the initialization of the partitions. In addition, we observe from Figure 7b,d that in K-means, the data points from the same cluster can be divided into separated clusters, and the points from different clusters can be clustered in a single cluster. All these mismatches will cause performance degradation in SLAM, which will be further demonstrated in Section 7.5.

### 7.4. Estimated Likelihoods

Given the channel estimation and the clustering performance, we now describe the obtained likelihood functions. For simplicity, we consider all dimensions independent and report the observed distributions of the number of paths and error of the specular path gain, delay and 4 angles. For diffuse paths, we report the distribution of the distances ${\hat{d}}^{i,l}$ and the channel gains. All these distributions are based on gathered channel estimation and clustering results in various vehicle locations. We use the setting in Section 7.1, and run the channel estimator many times, collect all channel estimation results and study statistics of collected data. Since the channel gains are strongly affected by path loss, (4)–(7) reduce the impact of the distance dependence by considering

(46) $$\begin{array}{c} {\overset{ˇ}{g}}^{i,l}\;\left[\mathrm{dB}\right]=20\log_{10}\left(|{\hat{g}}^{i,l}|∥x_{\mathrm{UE}}-x_{\mathrm{LM}}∥\frac{4\pi }{\lambda }\right). \end{array}$$

**Figure 8:**
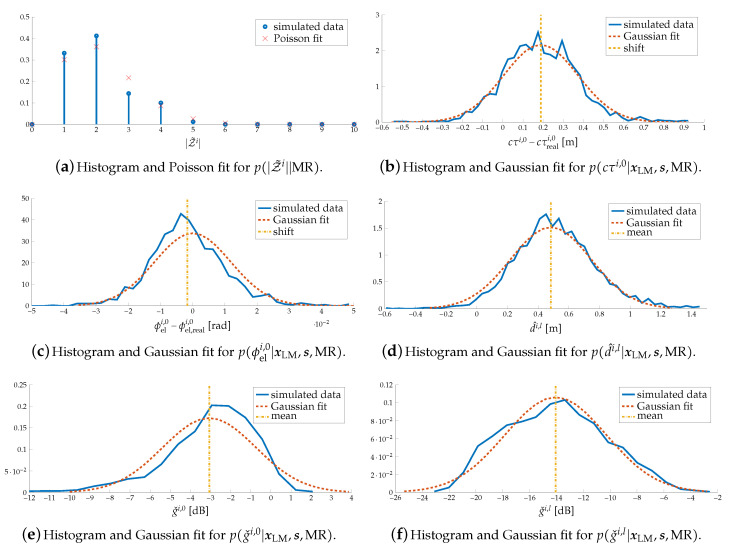
Some components of the likelihood for MR.

We first focus on the case m=MR as an example, and study $p(|{\tilde{Z}}^{i}||\mathrm{MR})$, $p(c\tau^{i,0}|x_{\mathrm{LM}},s,\mathrm{MR})$, $p(\phi_{\mathrm{el}}^{i,0}|x_{\mathrm{LM}},s,\mathrm{MR})$, $p({\overset{ˇ}{g}}^{i,0}|x_{\mathrm{LM}},s,\mathrm{MR})$, $p({\hat{d}}^{i,l}|x_{\mathrm{LM}},s,\mathrm{MR})$ and $p({\overset{ˇ}{g}}^{i,l>0}|x_{\mathrm{LM}},s,\mathrm{MR})$ in Figure 8. Figure 8a shows the histogram of the number of paths, as well as a Poisson approximation. We observe that there are always a (assumed) specular path and 1 to 5 diffuse paths, and the existing of more than one diffuse path follows a Poisson distribution. Figure 8b shows the histogram of the delay error of the first estimated path $\tau^{i,0}$ (i.e., subtracted with the delay of the specular path and multiple by the speed of light *c*) as well as a Gaussian fit. We observe that there is a shift of delay of 0.17 m, which is caused by inter-path interference. Figure 8c shows the histogram of the elevation AOD error of first estimated path $\phi_{\mathrm{el}}^{i,0}$ (i.e., subtracted with the angle of the specular path) as well as a Gaussian fit. We observe that inter-path interference does not influence the angle so much. Figure 8d shows the histogram and Gaussian fit of the distances ${\hat{d}}^{i,l},\;l>0$. As the inter-path interference leads to a positive delay shift, the estimated diffuse points are more likely to be behind the surface. Finally, Figure 8e,f shows the histogram of ${\overset{ˇ}{g}}^{i,0}$ and ${\overset{ˇ}{g}}^{i,l>0}$ as well as Gaussian fits. We observe that the specular path is stronger than diffuse paths, as the mean of ${\overset{ˇ}{g}}^{i,0}$ is larger, and diffuse paths are influenced by inter-path interference more seriously, as the distribution of ${\overset{ˇ}{g}}^{i,l>0}$ is more spread.

Table 3 provides a complete overview of the likelihood function for all landmark types. Based on all components of the likelihood functions for all landmarks, we have the following observations: for both cases m=BS and m=SM, there is always one path presents and all the angles, delays and ${\overset{ˇ}{g}}^{i,0}$ follow Gaussian distributions. For case m=MR, apart from the assumed specular path, there are 3 to 12 more paths present, and angles, delays, ${\overset{ˇ}{g}}^{i,l}$ and ${\hat{d}}^{i,l}$ also follow Gaussian distributions. We also find that the smoother surface has a higher gain for the specular path and lower gain for the diffuse path.

However, since the clustering algorithm is not perfect, it is possible that the cluster associated with a landmark may have fewer or more paths than the ground truth cluster. For example, the cluster associated with the MR may only have a specular path, due to the clustering error. We therefore set those possibility none zero in $p(|Z^{i}||m)$ manually, in order to increase the tolerance of the wrong clustering, as shown in Table 3.

### 7.5. SLAM Performance Evaluation

#### 7.5.1. Mapping Performance

Firstly, we study the performance of the proposed 5G SLAM scheme in mapping, conditioned on the true vehicle state. We use the generalized optimal subpattern assignment (GOSPA) distance [67] as the metric to evaluate the mapping result. The GOSPA metric considers the estimation error, which is the error between the estimated positions with the real positions of landmarks, the miss-detection error, which is the error of miss-detecting the existing landmarks, and the false alarm error, which is the error of detecting non-existing landmarks. The GOSPA between the estimate landmark position set $\hat{X}={\left\{{\hat{x}}_{i}\right\}}_{i=1}^{\hat{N}}$ and the real landmark position set $X={\left\{x_{j}\right\}}_{j=1}^{N}$ is defined as

(47) $$\begin{array}{cc} \; & d_{\mathrm{GOSPA}}\left(\hat{X},X\right)=\min_{\gamma \in \Gamma }{\left(\sum_{(i,j)\in \gamma }d^{q_{c}}\left({\hat{x}}_{i},x_{j}\right)+\frac{q_{c}^{q_{p}}}{q_{a}}\left(N_{\mathrm{miss}}^{\gamma }+N_{\mathrm{false}}^{\gamma }\right)\right)}^{\frac{1}{q_{p}}} \end{array}$$

where Γ is the set of all possible assignments; $N_{\mathrm{miss}}^{\gamma }$ is the number of miss detection of assignment γ; $N_{\mathrm{false}}^{\gamma }$ is the number of false alarm of assignment γ. The cardinality penalty factor $q_{a}$, the cut-off distance $q_{c}$, and the exponent factor $q_{p}$ are set as 20, 2, and 2, respectively.

We make two comparisons: (i) we compare different clustering methods (SLAM filter using modified DBSCAN which is the proposed method, DBSCAN without channel gains, and K-means); (ii) we compare SLAM filters with different numbers of paths (using all paths in every signal cluster based on the proposed likelihood function, which is the proposed method; using all paths but without using channel gains [24]; using the single (specular) path in every cluster based on the proposed likelihood function; using the single (specular) path in every cluster but without using channel gains, which is the PMBM extension of Reference [23]).

**Figure 9:**
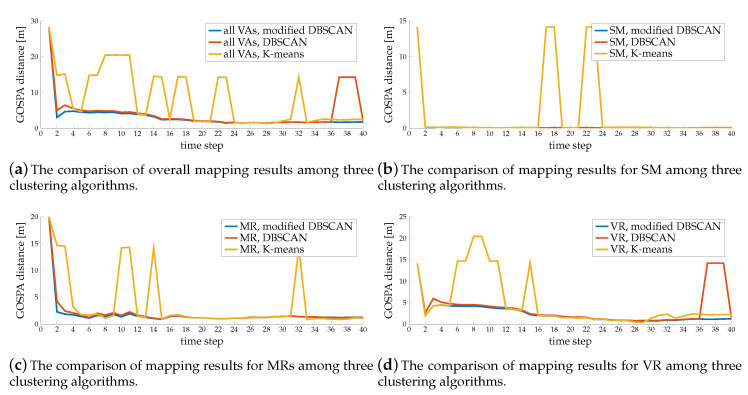
Comparison of GOSPA results among three clustering algorithms.

Figure 9 shows the outcome of the first comparison. We observe that the SLAM filter with K-means clustering performs poorly, even though the number of clusters is already known. Although the K-means performance in Table 2 seems good, grouping measurements from different sources leads to significant errors. The reason is that K-means only divides the space linearly and cannot handle complicated shapes. It often groups projected points from different sources into the same clusters; for example, at time step 17, it groups the measurements from BS and SM together. Modified DBSCAN and simple DBSCAN perform much better, as the blue and red lines are much lower, since both of them never group measurements from different sources together. The tensor ESPRIT channel estimator may create measurements that are far away from the specular path, and have low gains but still contain location information. Treating those outliers individually as single-element clusters in DBSCAN loses some information, and also forces the SLAM filter to create a new landmark, when similar outliers appear in successive time steps. This is apparent in the peak at time step 37–39 in Figure 9a, since there are some clutters pointing to a similar region between time step 36–39. With the help of the channel gains, the modified DBSCAN groups those measurements into the right clusters, which helps in mapping. That is the reason why the blue line is lower than the red line, for example at time step 2 in Figure 9c and time step 3 in Figure 9d.

**Figure 10:**
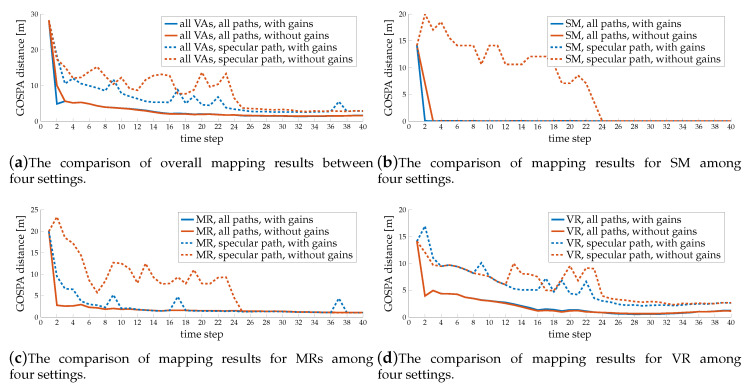
Comparison of GOSPA results among four different settings.

Figure 10 shows the comparison of the GOSPA results among 4 different settings (with or without using diffuse multipath, with or without using channel gain). We observe that SLAM filter has better performance when using diffuse multipath, as the solid lines are lower than the dashed lines, and it can also help the system to identify the landmark types, as there are no sharp peaks in solid lines, which is because more paths provide more information. We also observe the gain can improve the performance, especially in helping the SLAM filter to recognize the surface type, as the blue lines are lower than the red lines. Without information from channel gains, the SLAM filter has trouble to distinguish SM and MR when only using the specular path, as indicated by the sharp peaks of red dashed lines in Figure 10b,c and the red solid line dropping more slowly than the blue solid line in Figure 10b. This is because the distributions of delays and angles of specular paths from SM and MR are very close to each other, and some disturbance may cause a wrong identification. The information from channel gains will alleviate this problem. However, the channel gain is usually disturbed by inter-path interference, which increases with the roughness of the surface. Therefore, the gain is more informative for paths from SM and MR, but less informative for paths from VR, so the blue dashed lines are much lower than the red dashed lines in Figure 10b,c, but only slightly lower than the red dashed line in Figure 10d.

#### 7.5.2. Localization Performance

**Figure 11:**
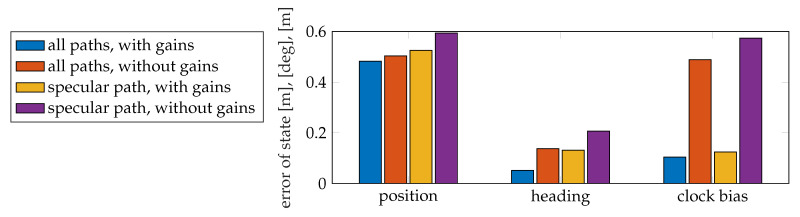
Comparison of vehicle state estimation performance, considering the utilization of channel gains and different sets of estimated propagation paths.

Next, we study the performance of the proposed 5G SLAM scheme in vehicle state estimation and compare the estimation results among the SLAM filter with different settings (with or without using diffuse multipath, with or without using channel gain). The landmarks are unknown and are mapped with tracking the vehicle state. We add ${\left[0.9\;m,0.9\;m,0m,4.5\;\deg,0m/s,0\mathrm{rad}/s,3\;\mathrm{ns}\right]}^{T}$ bias to the initial state, and the initial covariance is set as $\mathrm{diag}({\left[0.3\;m,0.3\;m,0\;m,0.1\;rad,0m/s,0\mathrm{rad}/s,1\;\mathrm{ns}\right]}^{2})$. We use 2000 particles to represent the vehicle state, and obtain the mean absolute error (MAE) between the real vehicle state and the estimated vehicle state after time step 5 until the simulation ends at time step 40

(48) $$\begin{array}{c} \mathrm{MAE}=\frac{\sum_{k=6}^{40}\left|s_{k}-{\hat{s}}_{k}\right|}{35}, \end{array}$$

as shown in Figure 11. The clock bias is multiplied with the speed of light *c* for evaluation. Overall, when using diffuse multipath, the SLAM filter has better performance in positioning, as MAEs are lower. The reason is that diffuse multipath provides more information than the single path. Using channel gain also improves the positioning performance, especially very helpful to estimate the clock bias, since gain contains information about propagation distance directly without being affected by the clock bias.

#### 7.5.3. Complexity Evaluation

**Figure 12:**
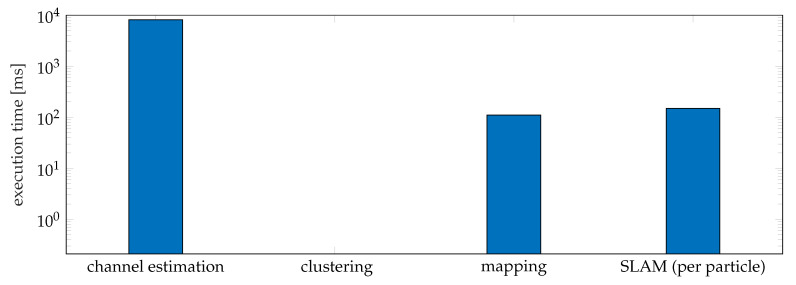
The execution times for each phase of the proposed framework.

We also evaluate the complexity of the proposed framework by measuring the execution time for each phase—channel estimation, clustering, mapping, SLAM (per particle), as shown in Figure 12. We measured a runtime per time step of 8.12 s for the channel estimation. The reason for taking a long time is that the ESPRIT channel estimator is based on a high dimensional tensor decomposition with high complexity. We measured the modified DBSCAN takes 0.21 ms per snapshot. For mapping, it takes 110 ms per time step. Although the proposed SLAM filter considers all possible data associations, as there are not too many landmarks under the simulation scenario, the number of all possible data associations is not large. However, the runtime will increase with more data associations. For SLAM, the proposed SLAM filter takes 149 ms per each particle (298.52 s in total) per time step. As 2000 particles are used and we need to fuse maps of all particles, the complexity is much higher than mapping, where the true vehicle state is given.

## 8. Conclusions

In this paper, we have treated the 5G SLAM problem from an end-to-end perspective, including downlink data transmission, channel estimation, clustering, and the SLAM filter. In the 5G SLAM problem, we aim to localize and synchronize a user while mapping the propagation environment, with the help of downlink signals from a single base station. We have proposed a novel method to cluster the MPCs by projecting the high-dimensional data into 3D points and then cluster the points based on the DBSCAN algorithm, which we augmented to account for the channel gains. We have also proposed a novel likelihood function in the 5G SLAM filter, which accounts for both the specular path as well as the diffuse multipath components.

Our results show that the ESPRIT channel estimator can estimate the channel parameters of both specular and diffuse multipath, and that the proposed system can directly use the raw un-clustered channel estimation results by applying the proposed clustering algorithms. With the help of the novel likelihood function, the proposed scheme can accurately estimate the number of landmarks, their types (i.e., roughness), and positions, and the channel gain is helpful in clustering and mapping and positing. The results also confirm that the proposed method can handle mapping and vehicle state estimation simultaneously, and highlight the benefit of considering both specular and diffuse multipath. In addition, the channel gains turn out the be highly informative for synchronizing the user to the base station.

The proposed framework has two computational bottleneck—the ESPRIT channel estimator and the particle filter used in the PMBM SLAM. In order to enable real-time execution, there is a need for faster solutions for both the channel estimation and the SLAM filter. The solutions could be either in the form of new algorithms, or by offloading the computation to more powerful edge computing systems, where edge servers can provide high-performance computing capability closer to end users [68,69,70].

## Figures and Tables

**Table 1:** Notations of important variables.

Notation	Definition	Notation	Definition
s	state of the user	$x_{\mathrm{LM}}$	landmark location
*m*	landmark type	θ	angle of arrival (AOA) pair
ϕ	angle of departure (AOD) pair	τ	time of arrival (TOA)
*g*	channel gain	Z	measurement set
z	single measurement	*c*	speed of light
$p_{D}$	detection probability	*k*	time index
*i*	surface index	l,p	path index
*s*	subcarrier index	*r*	dimension index

**Table 2:** Performance comparison of different clustering methods.

Clustering Method	Clustering Accuracy	Impurity
Modified DBSCAN	99.61%	0
DBSCAN	99.07%	0
K-means	94.63%	5.37%
Gap Statistics (GS)	68.64%	27.19%
Affinity Propagation (AP)	84.35%	12.54%

**Table 3:** Likelihood function for 5G SLAM for different types of landmarks.

Type *m*	$p(|{\tilde{Z}}^{i}||m)$	$p(\left[{\hat{d}}^{i,l};{\overset{ˇ}{g}}^{i,l}\right]|x_{\mathrm{LM}},s,m)$
BS	$\left(\right|{\tilde{Z}}^{i}{\left|-1\right)}^{*}∼0.9\Delta_{0}+0.1\;\mathrm{Geo}\left(0.9\right)$	N/A
SM	$|{\tilde{Z}}^{i}|∼0.9\Delta_{0}+0.1\;\mathrm{Geo}\left(0.9\right)$	N/A
MR	$\left(\right|{\tilde{Z}}^{i}\left|-1\right)∼0.1\Delta_{-1}+0.9\;\mathrm{Poi}\left(1.5\right)$	$N(\left[{\hat{d}}^{i,l};{\overset{ˇ}{g}}^{i,l}\right];\left[0.48;-14.1\right],\mathrm{diag}\left({\left[0.35,3.8\right]}^{2}\right)$
VR	$\left(\right|{\tilde{Z}}^{i}\left|-3\right)∼0.1\sum_{n=1}^{3}0.5^{n-1}\Delta_{-n}+0.825\;\mathrm{Geo}\left(0.27\right)$	$N(\left[{\hat{d}}^{i,l};{\overset{ˇ}{g}}^{i,l}\right];\left[0.48;-9.4\right],\mathrm{diag}\left({\left[0.35,3.8\right]}^{2}\right)$
	$p({\overset{ˇ}{z}}^{i,0}|x_{\mathrm{LM}},s,m)$	
BS	$N({\overset{ˇ}{z}}^{i,0};\left[h_{\mathrm{VA}};0\right],\mathrm{diag}\left({\left[0.003,0.0001\times 1_{4},0.005\right]}^{2}\right))$	
SM	$N({\overset{ˇ}{z}}^{i,0};\left[h_{\mathrm{VA}};-1.93\right],\mathrm{diag}\left({\left[0.01,0.002\times 1_{4},0.02\right]}^{2}\right))$	
MR	$N({\overset{ˇ}{z}}^{i,0};\left[h_{\mathrm{VA}};-3.1\right]+{\left[0.17,0_{1\times 5}\right]}^{T},\mathrm{diag}\left({\left[0.19,0.012\times 1_{4},2.3\right]}^{2}\right))$	
VR	$N({\overset{ˇ}{z}}^{i,0};\left[h_{\mathrm{VA}};-6.3\right]+{\left[0.8,0_{1\times 5}\right]}^{T},\mathrm{diag}\left({\left[0.5,0.05\times 1_{4},3.8\right]}^{2}\right))$	

* $\left|Z\right|-M∼p\Delta_{n}+\left(1-p\right)\;\mathrm{Geo}\left(\gamma \right)$ means that |Z| is equal to *M* plus a random variable drawn from a discrete mixture distribution with two components: one with mass *p* at value *n* and one that is a scaled Geometric distribution (with scaling 1−p). Poi(γ) represents a Poisson distribution. ${\overset{ˇ}{z}}^{i,0}$ denotes $\left[c\tau^{i,0};\theta^{i,0};\phi^{i,0};{\overset{ˇ}{g}}^{i,0}\left[\mathrm{dB}\right]\right]$. $h_{\mathrm{BS}}$, $h_{\mathrm{VA}}$ are the geometric relations $h(x_{\mathrm{BS}},s)$ and $h(x_{\mathrm{VA}},s)$, which can be found in Appendix A.

## Appendix A. Geometric Relations

The geometric relations are as follows. For LOS, $h(x_{\mathrm{BS}},s)$ returns

- TOA: $\tau =∥x_{\mathrm{UE}}-x_{\mathrm{BS}}∥/c+B.$
- AOD pair: $\phi_{\mathrm{az}}=\arctan\left(y_{\mathrm{UE}}-y_{\mathrm{BS}},x_{\mathrm{UE}}-x_{\mathrm{BS}}\right)$, $\phi_{\mathrm{el}}=\arcsin\left(\left(z_{\mathrm{UE}}-z_{\mathrm{BS}}\right)/∥x_{\mathrm{UE}}-x_{\mathrm{BS}}∥\right).$
- AOA pair: $\theta_{\mathrm{az}}=\arctan\left(y_{\mathrm{BS}}-y_{\mathrm{UE}},x_{\mathrm{BS}}-x_{\mathrm{UE}}\right)+\pi -\varpi$, $\theta_{\mathrm{el}}=\arcsin\left(\left(z_{\mathrm{BS}}-z_{\mathrm{UE}}\right)/∥x_{\mathrm{UE}}-x_{\mathrm{BS}}∥\right).$

For the case of a specular component, $h(x_{\mathrm{VA}},s)$ returns

- TOA: $\tau =∥x_{\mathrm{VA}}-x_{\mathrm{UE}}∥/c+B.$
- AOD pair: $\phi_{\mathrm{az}}=\arctan\left(y_{\mathrm{sp}}-y_{\mathrm{BS}},x_{\mathrm{sp}}-x_{\mathrm{BS}}\right)$, $\phi_{\mathrm{el}}=\arcsin\left(\left(z_{\mathrm{sp}}-z_{\mathrm{BS}}\right)/∥x_{\mathrm{sp}}-x_{\mathrm{BS}}∥\right).$
- AOA pair: $\theta_{\mathrm{az}}=\arctan\left(y_{\mathrm{VA}}-y_{\mathrm{UE}},x_{\mathrm{VA}}-x_{\mathrm{UE}}\right)-\varpi$, $\theta_{\mathrm{el}}=\arcsin\left(\left(z_{\mathrm{VA}}-z_{\mathrm{UE}}\right)/∥x_{\mathrm{VA}}-x_{\mathrm{BS}}∥\right)$.

where $x_{\mathrm{sp}}$ is the incident point, which can be calculated by (31). For the case of a diffuse component with diffuse point $x_{\mathrm{sc}}$, $h(x_{\mathrm{sc}},s)$ returns

- TOA: $\tau =(∥x_{\mathrm{BS}}-x_{\mathrm{sc}}∥+∥x_{\mathrm{sc}}-x_{\mathrm{UE}}∥)/c+B.$
- AOD pair: $\phi_{\mathrm{az}}=\arctan\left(y_{\mathrm{sc}}-y_{\mathrm{BS}},x_{\mathrm{sc}}-x_{\mathrm{BS}}\right)$, $\phi_{\mathrm{el}}=\arcsin\left(\left(z_{\mathrm{sc}}-z_{\mathrm{BS}}\right)/∥x_{\mathrm{sc}}-x_{\mathrm{BS}}∥\right).$
- AOA pair: $\theta_{\mathrm{az}}=\arctan\left(y_{\mathrm{sc}}-y_{\mathrm{UE}},x_{\mathrm{sc}}-x_{\mathrm{UE}}\right)-\varpi$, $\theta_{\mathrm{el}}=\arcsin\left(\left(z_{\mathrm{sc}}-z_{\mathrm{UE}}\right)/∥x_{\mathrm{sc}}-x_{\mathrm{BS}}∥\right)$.

## Appendix B. PMBM SLAM Filter

### Appendix B.1. Representation of PMBM Density

We adopt the finite set statistic (FISST) for the multi-target tracking [71], and we introduce RFS X for a multi-target state, which consists of the undetected targets $X_{U}$ that have never been detected before and detected targets $X_{D}$ that have been detected at least once. The PMBM density is denoted by [65]

(A1) $$f\left(X\right)=\sum_{X_{U}⊎X_{D}=X}f_{P}\left(X_{U}\right)f_{\mathrm{MBM}}\left(X_{D}\right),$$

where ⊎ denotes the union of mutually disjoint sets, $f_{P}\left(\cdot \right)$ denotes a Poisson point process (PPP) density, and $f_{\mathrm{MBM}}\left(\cdot \right)$ denotes an multi-Bernoulli mixture (MBM) density. The PPP density $f_{P}\left(X_{U}\right)$ is defined as

(A2) $$f_{P}\left(X_{U}\right)=e^{-\int \lambda (x)dx}\prod_{j=1}^{n}\lambda \left(x^{j}\right),$$

where λ(·) is the intensity function called as probability hypotheses density [72], *n* is the cardinality of $X_{U}$. The MBM density $f_{\mathrm{MBM}}\left(X_{D}\right)$ is represented as

(A3) $$f_{\mathrm{MBM}}\left(X_{D}\right)\propto \sum_{h}\sum_{X^{1}⊎\cdots ⊎X^{n}=X_{D}}\prod_{j=1}^{n}l^{h,j}f_{B}^{h,j}\left(X^{j}\right),$$

where *h* indicates the hypotheses index [18], *n* is the number of potentially detected objects, and $f_{B}^{h,j}\left(\cdot \right)$ denotes the Bernoulli density of the object *j* under the global hypothesis *h*, and $l^{h,j}$ is its weight. Each Bernoulli density follows

(A4) $$f_{B}^{h,j}\left(X^{j}\right)=\left\{\begin{array}{cc} 1-r^{h,j}\; & X^{j}=\emptyset \\ r^{h,j}f^{h,j}\left(x\right)\; & X^{j}=\left\{x\right\}\\ 0\; & \mathrm{otherwise} \end{array}\right.$$

where $r^{h,j}$ is the existence probability and $f^{h,j}\left(\cdot \right)$ is the state density. Then, (A2) can be parameterized by λ(x), and (A3) can be parameterized by ${\left\{{\left\{l^{h,j},r^{h,j},f^{h,j}\left(x\right)\right\}}_{j\in I^{h}}\right\}}_{h\in I}$, where I is the index set.

### Appendix B.2. Implementation of PMBM SLAM Filter

The PMBM SLAM filter goes through prediction and update steps of the Bayesian filtering recursion as in [23]. For implementation, we adopt the Rao-Blackwellized approach; particles represent the user state, and PMBM densities conditioned on each particle indicate the landmarks. At the beginning of time *k*, ${\left\{s_{0:k-1}^{n},\omega_{k-1|k-1}^{n}\right\}}_{n=1}^{N}$ is given, where $\omega_{k-1|k-1}^{n}\geq 0$ and $\sum_{n}\omega_{k-1|k-1}^{n}=1$, and we have a PMBM density with PPP parameter $\lambda_{k-1|k-1}^{n}\left(x\right)$ and MBM parameters ${\left\{{\left\{l_{k-1|k-1}^{n,h,j},r_{k-1|k-1}^{n,h,j},f_{k-1|k-1}^{n,h,j}\left(x\right)\right\}}_{j\in I_{k}^{n,h}}\right\}}_{h\in I_{k-1}^{n}}$ for all particle *n*. For notation simplicity, we omit the particle index *n* in the PPP and MBM parameters.

1. *User Prediction*: Using (1), the user particle $s_{k-1|k-1}^{n}$ is predicted as $s_{k|k-1}^{n}=v\left(s_{k-1|k-1}^{n}\right)+q_{k}^{n}$, where $q_{k}^{n}∼N\left(0,Q_{k}\right)$ and $\omega_{k|k-1}^{n}=\omega_{k-1|k-1}^{n}$.
2. *Map Prediction*: Since the targets are static, the PPP parameter is predicted as $\lambda_{k|k-1}\left(x\right)=p_{S}\lambda_{k-1|k-1}\left(x\right)+\lambda_{B,k}\left(x\right)$, where $p_{S}$ is the survival probability, $\lambda_{B,k}\left(x\right)$ is the birth intensity. For the MBM components, $l_{k|k-1}^{h,j}=l_{k-1|k-1}^{h,j}$, $r_{k|k-1}^{h,j}=p_{S}r_{k-1|k-1}^{h,j}$, and $f_{k|k-1}^{h,j}\left(x\right)=f_{k-1|k-1}^{h,j}\left(x\right)$.
3. *Map Update*: The map update is divided into the following four cases [65]
  (a) *Missed detections for undetected objects:* The undetected objects remain as the undetected objects, and thus is given by
    (A5) $$\begin{array}{c} \lambda_{k|k}\left(x\right)=\left(1-p_{D}\right)\lambda_{k|k-1}\left(x\right), \end{array}$$
    where $p_{D}$ indicates the detection probability.
  (b) *Detections for the first time:* Using the grouped measurement $Z_{k}^{i}$ and the PPP parameter $\lambda_{k|k-1}\left(x\right)$, we newly generate the MBM parameters as
    (A6) $$\begin{array}{cc} \; & r_{U,k|k}^{i}=\rho_{U,k|k-1}^{i}\left(Z_{k}^{i}\right)/\left(c\left(Z_{k}^{i}\right)+\rho_{U,k|k-1}^{i}\left(Z_{k}^{i}\right)\right), \end{array}$$
    (A7) $$\begin{array}{cc} \; & f_{U,k|k}^{i}\left(x\right)=\ell \left(Z_{k}^{i}|x,s_{k|k-1}\right)\lambda_{k|k-1}\left(x\right)/\rho_{U,k|k-1}^{i}\left(Z_{k}^{i}\right), \end{array}$$
    (A8) $$\begin{array}{cc} \; & l_{U,k|k}^{i}=c\left(Z_{k}^{i}\right)+\rho_{U,k|k-1}^{i}\left(Z_{k}^{i}\right), \end{array}$$
    (A9) $$\begin{array}{cc} \; & \rho_{U,k|k-1}^{i}\left(Z_{k}^{i}\right)=\int \ell \left(Z_{k}^{i}|x,s_{k|k-1}\right)\lambda_{k|k-1}\left(x\right)dx, \end{array}$$
    where $c\left(Z_{k}^{i}\right)=\delta (|Z_{k}^{i}\left|=1\right)c\left(z_{k}^{i,0}\right)$.
  (c) *Missed detections for the previously detected objects:* The detected objects also remain as the detected objects, and then the MBM parameters have no measurement update
    (A10) $$\begin{array}{cc} \; & r_{k|k}^{h,j,0}=\left(1-p_{D}\right)r_{k|k-1}^{h,j}/\left(1-p_{D}r_{k|k-1}^{h,j}\right), \end{array}$$
    (A11) $$\begin{array}{cc} \; & f_{k|k}^{h,j,0}\left(x\right)=f_{k|k-1}^{h,j}\left(x\right), \end{array}$$
    (A12) $$\begin{array}{cc} \; & l_{k|k}^{h,j,0}=l_{k|k-1}^{h,j}\left(1-r_{k|k-1}^{h,j}p_{D}\right). \end{array}$$
  (d) *Detections for the previously detected objects*: Using $Z_{k}^{i}$, the MBM parameters are computed as
    (A13) $$\begin{array}{cc} \; & r_{k|k}^{h,j,i}=1, \end{array}$$
    (A14) $$\begin{array}{cc} \; & f_{k|k}^{h,j,i}\left(x\right)=\ell \left(Z_{k}^{i}|x,s_{k|k-1}\right)f_{k|k-1}^{h,j}\left(x\right)/\rho_{k|k-1}^{h,j,i}\left(Z_{k}^{i}\right), \end{array}$$
    (A15) $$\begin{array}{cc} \; & l_{k|k}^{h,j,i}=l_{k|k-1}^{h,j}r_{k|k-1}^{h,j}\rho_{k|k-1}^{h,j,i}\left(Z_{k}^{i}\right), \end{array}$$
    (A16) $$\begin{array}{cc} \; & \rho_{k|k-1}^{h,j,i}\left(Z_{k}^{i}\right)=\int \ell \left(Z_{k}^{i}|x,s_{k|k-1}\right)f_{k|k-1}^{h,j}\left(x\right)dx. \end{array}$$
  To reduce the exponentially increasing data association, we adopt the pruning and merging process [65] with the Murty’s algorithm [73], where the cost matrix is generated by $l_{U,k|k}^{i}$, $l_{k|k}^{h,j,0}$ and $l_{k|k}^{h,j,i}$ calculated in (A8), (A12) and (A15).
4. *User Update*: Each particle weight is updated as
  (A17) $$\omega_{k|k}^{n}\propto \omega_{k|k}^{n}\sum_{h}l_{k|k}^{n,h},$$
  where $l_{k|k}^{n,h}$ is the weight for the updated global hypothesis *h* and particle *n*. Then, the user state is estimated by ${\hat{s}}_{k|k}=\sum_{n}\omega_{k|k}^{n}s_{k|k}^{n}$.

Given the number of particles *N*, the user state prediction requires $O\left(N\right)$ computations. For each particle, the map prediction requires $O\left(N_{B}\right)$ computations (if there are $N_{B}$ unique Bernoulli components for the given particle), and the map update process requires $O\left(N_{B}\hat{I}\right)$ computations for updating Bernoulli components and $O\left(N_{B}N_{G}\sum_{t=0}^{\hat{I}}C_{\hat{I}}^{t}A_{N_{B}}^{\hat{I}-t}\right)$ for updating data associations and their weights (if there are $\hat{I}$ measurement clusters and $N_{G}$ global hypotheses in total for the given particle), where *A* represents the permutation operation and *C* represents the combination operation. The complexity of the particle weight updating is $O\left(N_{G}\right)$ for each particle, and the complexity of state estimation is $O\left(N\right)$. Note: each particle may have different $N_{G}$ and $N_{B}$.

### Appendix B.3. Map Fusion

We need to create a map using these particles. Since there is a different MBM for each particle, and an MB represents a possible map, a best map needs to be computed. Firstly, we need to compute an MB for each particle. The MBM with parameters ${\left\{{\left\{l_{k|k}^{n,h,j},r_{k|k}^{n,h,j},f_{k|k}^{n,h,j}\left(x\right)\right\}}_{j\in I_{k}^{n,h}}\right\}}_{h\in I_{k}^{n}}$ can be written as ${\left\{q_{k|k}^{n,h},{\left\{r_{k|k}^{n,h,j},f_{k|k}^{n,h,j}\left(x\right)\right\}}_{j\in I_{k}^{n,h}}\right\}}_{h\in I_{k}^{n}}$, where $q_{k|k}^{n,h}$ is the probability of the global hypothesis *h*, which follows

(A18) $$\begin{array}{c} q_{k|k}^{n,h}\propto \prod_{j\in I_{k}^{n,h}}l^{n,h,j}. \end{array}$$

The existence probability of each new MB component will be [18]

(A19) $$\begin{array}{c} {\bar{r}}_{k|k}^{n,j}=\sum_{h\in I_{k}^{n}}q_{k|k}^{n,h}r_{k|k}^{n,h,j}, \end{array}$$

and the density becomes

(A20) $$\begin{array}{c} {\bar{f}}_{k|k}^{n,j}\left(x\right)=\frac{\sum_{h\in I_{k}^{n}}q_{k|k}^{n,h}f_{k|k}^{n,h,j}\left(x\right)}{{\bar{r}}_{k|k}^{n,j}}. \end{array}$$

Then, we have an MB with parameters ${\left\{{\bar{r}}_{k|k}^{n,j},{\bar{f}}_{k|k}^{n,j}\left(x\right)\right\}}_{j\in {\bar{I}}_{k}^{n}}$ for particle *n*. When considering all particles, we would create a larger MB by concatenating all MBs of different particles together, with parameters ${\left\{{\left\{{\bar{r}}_{k|k}^{n,j}\omega_{k|k}^{n},{\bar{f}}_{k|k}^{n,j}\left(x\right)\right\}}_{j\in {\bar{I}}_{k}^{n}}\right\}}_{n\in I_{k}}$. Finally, we merge components with Euclidean distances smaller than the given threshold in the new MB, and the finial MB is the best map. The map fusion requires $O\left(NN_{G}N_{B}+{\left(NN_{B}\right)}^{2}\right)$ computations, when considering the merging process.
